# mORCA: ubiquitous access to life science web services

**DOI:** 10.1186/s12864-018-4439-x

**Published:** 2018-01-16

**Authors:** Sergio Diaz-del-Pino, Oswaldo Trelles, Juan Falgueras

**Affiliations:** 1 Computer Architecture Department, University of Malaga, Bulevar Luis Pasteur 35, 29071 Malaga, Spain; 2 Computer Languages and Computer Science Department, University of Malaga, Bulevar Luis Pasteur 35, 29071 Malaga, Spain

**Keywords:** Bioinformatics, Biomedicine, Mobile-devices, Software clients, Web services

## Abstract

**Background:**

Technical advances in mobile devices such as smartphones and tablets have produced an extraordinary increase in their use around the world and have become part of our daily lives. The possibility of carrying these devices in a pocket, particularly mobile phones, has enabled ubiquitous access to Internet resources. Furthermore, in the life sciences world there has been a vast proliferation of data types and services that finish as Web Services. This suggests the need for research into mobile clients to deal with life sciences applications for effective usage and exploitation.

**Results:**

Analysing the current features in existing bioinformatics applications managing Web Services, we have devised, implemented, and deployed an easy-to-use web-based lightweight mobile client. This client is able to browse, select, compose parameters, invoke, and monitor the execution of Web Services stored in catalogues or central repositories. The client is also able to deal with huge amounts of data between external storage mounts. In addition, we also present a validation use case, which illustrates the usage of the application while executing, monitoring, and exploring the results of a registered workflow. The software its available in the Apple Store and Android Market and the source code is publicly available in Github.

**Conclusions:**

Mobile devices are becoming increasingly important in the scientific world due to their strong potential impact on scientific applications.

Bioinformatics should not fall behind this trend. We present an original software client that deals with the intrinsic limitations of such devices and propose different guidelines to provide location-independent access to computational resources in bioinformatics and biomedicine. Its modular design makes it easily expandable with the inclusion of new repositories, tools, types of visualization, etc.

**Electronic supplementary material:**

The online version of this article (10.1186/s12864-018-4439-x) contains supplementary material, which is available to authorized users.

## Background

It is commonplace to say that bioinformatics and biomedicine (BIBM) applications are mostly deployed in the Web. The biggest contributors in this setting (e.g. EBI [1], NCBI [2], and INB [3]), offer web access to databases and data analysis applications served by their computing infrastructures via different interfaces.

Furthermore, mobile devices have experienced a continuous increase in popularity and availability. This increase has motivated the implementation of new clients for these devices to access scientific applications. Such clients could be potentially useful in multiple scenarios, from checking executions and rerun in case of error, to the use of teaching or divulgation purpose in classroom or a conference.

Given that the majority of mobile devices have a web-browser, it is fair to assume an easy adaptation of current BIBM web-based software clients to such devices. Nevertheless, in practice such adaptation is not a simple and direct procedure. This adaptation process necessitates a profound review of the capabilities of these new devices in order to decide the correct manner to replicate the functionality of already available software for desktop computers and laptops.

Several useful BIBM tools have already been revised for mobile devices, including Biocatalogue [4], SimAlign [5], and ‘Oh BLAST it!’ [6] (see a detailed list in Additional file 1). An unreasonable amount of effort would be required to build a different application to adapt every piece of BIBM software. Therefore, we have devised a more general strategy to avoid one-by-one service migration, by using repositories in which service metadata (e.g. the service’s name, input/output parameters, and description) are stored. This facilitates the incorporation of new services and enables the production of uniform user-friendly interfaces. The system should be easily expandable such that it can incorporate new tools. Thus, we start by defining the needed functionality intrinsic to this kind of system.

### Considerations regarding mobile applications and interfaces

There are significant differences between the traditional WIMP (Windows, Icons, Menus, and Pointer) interfaces and mobile (or touch user) interfaces (GUI). Details such as the replacement of mouse hovering effects with specific touch gestures and the use of multitouch have to be considered [7]. The high number of changes suggested a total redesign of the interaction style.

In the particular case of BIBM applications, we have to take into account considerations regarding distraction issues and the display of clear and appropriate information to make them suitable in complex settings [8]. These issues mean that the application must allow rapid handling, easy interaction, and accurate and rapid reading. It must also avoid clutter, especially in the display of BIBM application results [9, 10]. These issues require applying Human Computer Interaction methods from design through procurement, training, and use [11].

In the emerging area of mobile application development, there still are few standards to follow. The rapid expansion of mobile devices, which is expected to reach 2 billion users worldwide in 2019 [12] is hindering the establishment of specific development models. Nevertheless, mobile applications development can be classified into different programming models [13]:

- **Native applications** are implemented using only the programming language designed for the device. These applications can only be executed in the platform it was developed for, but they can use every internal feature of the device (GPS, accelerometer, contacts, calendar, etc). Users appreciate them because of their integration and efficiency. However, this approach requires maintaining specific applications for each type of device operating system, which translates into the development of specific applications for each platform with the consequent increase in development time and budget.
- **Web-based applications** can be executed on any platform simply requiring a mobile web-browser. Important drawbacks include slowness, limited functionality and performance, and total dependence on the Web. However, they can access mobile information, such as persistent data, or geolocation through HTML5-specific methods.
- The two prior models combine to form **hybrid applications**. They provide applications created with Web technologies that run natively on the device as a web container. Native applications provide some of the advantages of this model (e.g., access to the device’s APIs, Stores distribution, and offline mode). All the previous statements combined with a cross-platform development provided by the use of Web technologies. Hybrid applications also have some disadvantages, most of which are related to performance requirements or user experience (UX) design, which has been dramatically improved in recent years.

### Catalogues of BIBM web services: Browsing, discovering and invocation

In the extraordinarily active BIBM research field, there is continuous growth in the already high number of available software as novel tools, demands, and data types emerge (e.g. over 2000 services are registered in Bio.tools [14], which is the most recent attempt to create a BIBM catalogue). This considerable amount of resources motivates the creation of an intelligent software organisation to make easier the integrated use of tools. The final aims are to remove the necessity of building custom interfaces for tools and to assist the users to discover suitable tools for their analyses.

Centralized repositories emerge as the preferred choice to categorise service metadata, such as service description, parameters, data types, and documentation. At present, there exist a number of these metadata catalogues in the BIBM fields, such as BioCatalogue [4], which is one of the most representative for bioinformatics Web Services, and Bio.tools, which appeared recently as a community effort initiated by the ELIXIR project to document tools and data services in bioinformatics.

The invocation of a service begins with the composition of its interface of parameters, which is manually or automatically designed using the parameter descriptions available in the repositories. The user then fills the requested parameters and launches the service. Web Services can be executed synchronously or asynchronously. The synchronous invocation of services blocks the interface until execution is finished and the results can be consulted, whereas asynchronous calls proceed without blocking. Asynchronous calls are the most appropriate, given the intrinsic characteristics of these BIBM services, which use large amounts of data and could entail long processing times.

Several tools are available for registering, browsing, discovering, and invoking services from different metadata repositories (e.g. jORCA [15], MOWServ [16], Taverna [17], Seahawk [18], and EMBL-EBI [19]). Overall, existing solutions target only personal computers (i.e. the platforms that were available when they were implemented). Such solutions are inappropriate for mobile devices, which have a limited screen size and a different way of user interaction. For instance, looking for a service can be a challenge, especially on repositories with a large list of them. Exploring the complete resources tree while seeking for a given item might be a laborious and time-consuming task. Additionally, parameter selection and data entry is performed interacting with the screen rather than by a mouse-click. In fact, mobile devices pose a real challenge to what are new forms of interaction, data sharing between apps, and storing and accessing the results.

### Additional functionality

The most common functions of BIBM clients (i.e. registering, browsing, discovering, and executing services) should be extended with the following features:

- **Data management**: Exploring, creating, and deleting files are mandatory operations. The system also requires a component to enable users to upload and download their files to/from the system. The current bigdata world still faces challenges related to data transfer. Decoupling service invocation from data upload is required to avoid data transfer problems during invocations and file re-transmission.
- **User account management**: The system also requires a component to manage user authentication, privileges, and data privacy to protect sensitive data.
- **Multi-repository management**: As mentioned, repositories store service metadata. However, due to the lack of standardization and maturity, new and different releases are coming out in an attempt to find the best solution. These solutions usually have points in common (e.g service description and parameters) that can be mapped to a common metadata representation.

## Implementation

The presented application (mORCA) significantly extends the functionality of the initial mobile client prototype described in our previous work [20] In this new development we have included important new features such as asynchronous calls with monitoring capabilities, the evolution from web-app to hybrid app, or the development of new modules to list and execute third-party services. Additionally, minor changes have been done including a new fuzzy search mechanism, a user accounting module, and a complete restyling of the application after performing a usability study with users of the BIBM application domains.

In this section the design and implementation of a mobile client satisfying such requirements is outlined. The current capabilities of mobile devices have been considered during the whole life cycle.

### Architecture

Our design consists of a server-side in which MAPI and its Web Service are in charge of communicating with both the repositories and the client. The client-side deals with the information provided by the server through different modules and also it manages how this information is shown to the user.

**Fig. 1:**
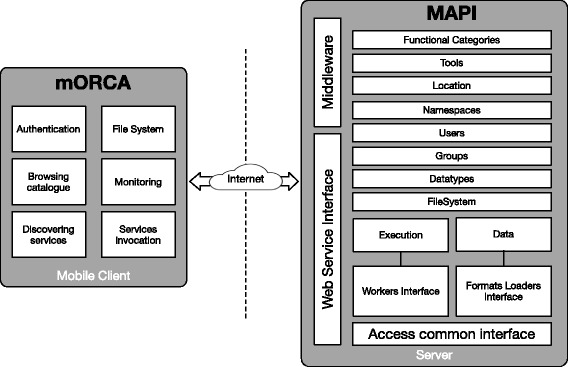
System Architecture. The modular application uses a Web Service to connect with the mAPI framework, which is in the server-side and covers functionality related to service-oriented architectures, in particular, management of metadata for WS tools and categories, data types, data, files, and users

MAPI [21] is a tool for the standardization of the metadata contained in different Web Service repositories. It permits the use of different formats of services using a standard interface in a transparent way since the data type module manages these different formats. In addition, MAPI enables the execution of services responding to diverse communication protocols (e.g. SOAP, REST, and BioMOBY [22]). To perform this task, it implements a set of execution workers (i.e. service invocation modules), which are specifically designed to execute a given type of service. Further functionality includes user management, and file system operations (see the MAPI modules represented as boxes in the right part of Fig. 1).

The modules of the client-side are in charge of the main functionality described before:

- User authentication
- Browsing the repository
- Discovering services
- Service parameter composition
- Service invocation
- File management, including data uploading and downloading
- Service execution monitoring

### Software specifications

This section addresses the technologies we used to implement our software. It has been split into two subsections: the client-side and the server-side.

#### Client-side

In *Considerations regarding mobile applications and interfaces*, we decided to develop a hybrid application. This kind of mobile development allowed us to focus on the application, whatever the platform, and to achieve the performance and functionality requirements of our setup. These requirements have motivated a complete restyling.

Web technologies such as jQuery Mobile [23] were used to make the interface responsive to a wide variety of screen sizes and to adapt the data input to the devices (e.g numerical keyboard and drop-down list). jQuery [24] in combination with VanillaJS were used to implement the main functionality and handlers for the backend petitions. The software has been packed using Cordova [25] in order to make it compatible with the Apple Store and the Google Market.

#### Server-side

We developed two layers to access MAPI in our server-side (see Fig. 1). The first is a Web Service layer to access most of the methods provided by MAPI. This Web Service is developed in Java, runs in a Tomcat container, and uses the SOAP protocol to communicate with the client.

The second layer is a small middleware developed for the service monitoring module using NodeJS [26] and MongoDB [27] to track and store user executions.

### Main functionality

#### Browsing the catalogue

**Fig. 2:**
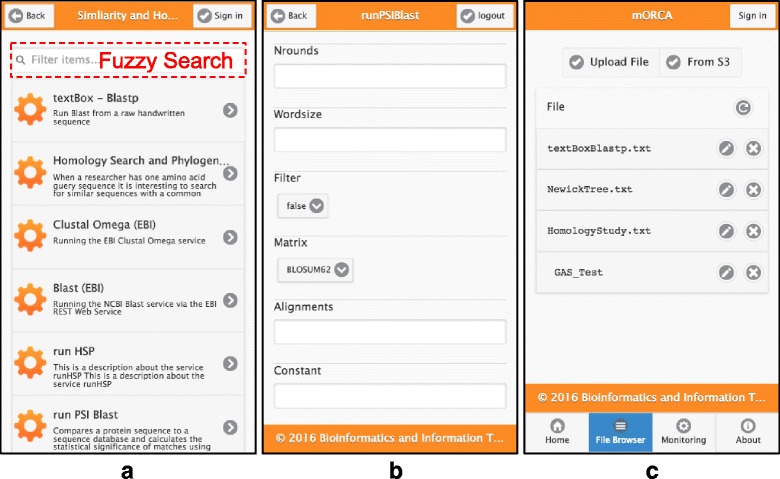
**a** The nested “Listview” used to represent hierarchical trees in mobile devices. **b** The File system view with the main folder of the guest user with common CRUD operations. **c** A dynamically generated interface for the EBI BLAST service, which is one of the most common services in bioinformatics

Access to Web Service repositories is delegated to the server-side. The server-side is able to work with multiple repositories by implementing different accesses for each of the configured catalogues, decoupling the client-side from this task. The client- side just requests a hierarchical list of services from the server-side. To display the retrieved service tree, whatever the catalogue, a nested “Listview” was used to represent it as a list of folders and services (see Fig. 2a). This representation enables users to do a fast examination of the Web Services tree. Later on users can look for a specific service by navigating through the categories. During the navigation process, new panels with the services and subcategories of the selected category appear from the right-hand side.

Using this implementation, the developed mobile client is able to browse all the service catalogues that the MAPI server-side is able to handle. This includes the traditional BioMOBY repositories of the Spanish National Institute of Bioinformatics, and other catalogues such as Bio.tools and BioCatalogue. New catalogues can be easily added to the system by mapping its service metadata to our data structure.

Since a given catalogue can potentially contain a huge number of services, a textbox component (see Fig. 2a) provides users the possibility to filter the services tree according to a user-defined search criteria. The search engine developed, which lists just the corresponding services and categories, is a simplification of the service discovery function of the Magallanes tool [28]. It is important to note that the implemented search works in a fuzzy way, meaning that it produces results even if the search criteria lead to some mismatches.

#### Service invocation and monitoring

The client invokes the Web Services using the execution workers present in the MAPI modules of the server-side, which enable executing proprietary and third- party services. This service invocation is performed in an asynchronous way given the large execution times reported by most existing services.

**Fig. 3:**
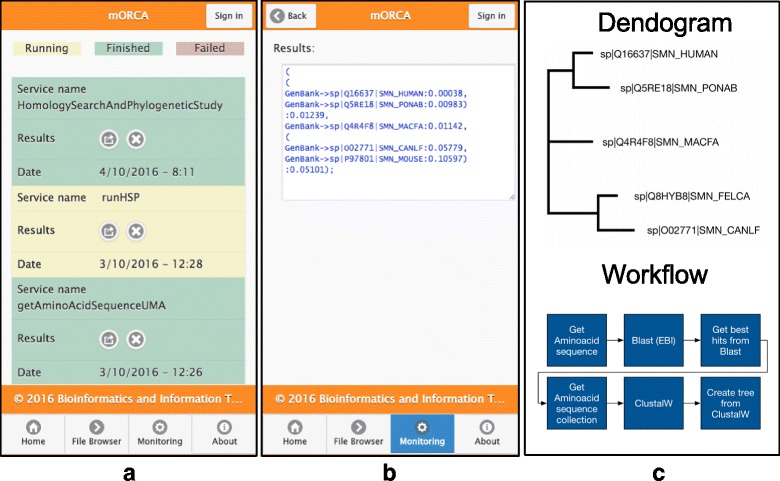
**a** The monitoring interface that shows two finished services and one running; **b** The results of running the ‘homology search and phylogenetic study’ workflow described in the guided exercise; **c** The results shown as a sketched dendogram and the representation of the workflow

Firstly, the service execution interface is dynamically generated with the information retrieved from the metadata repository (see Fig. 2b). Next, before invoking the service, the user has to fill out the input parameters and the output filename. Files can also be selected from a list of remotely stored files for the input parameters that accept it. This is done to remove the input data from the invocation payload, as was traditionally carried out in most traditional executions (i.e. BioMoby). Parameters are filled with the default values stored in the repository. After filling the parameters the service can be executed. Once invoked, the status of the service can be checked in the monitoring section. In this section, there is another “Listview” with information about the service and a colour representation of status (see Fig. 3a), which is automatically refreshed when a service finishes the task. By applying a criterion similar to that used for the input file, the service output is saved in the server-side data storage. We provide different approaches to retrieve the output file from remote storage.

#### File system

The mobile application uses the file system component of MAPI, which is located on the server-side, through its Web Service interface. The file system component offers the usual file system operations such as browsing directories or creating and deleting files. All the previously mentioned operations are performed in the server side by MAPI. The implemented file system does not store data on the client-side due to the size of nowadays bioinformatics datasets and to avoid file re-transmission in each service execution. The file system uses the user accounting system to store the data in folders separated by users in the server-side.

Different options are provided for data uploading and downloading, apart from using the server-side uploading mechanism. These alternatives include plugins to import data to the server-side data storage using Globus Online [29] or Amazon S3 [30]. Amazon S3 can be accessed through the client, allowing the user to import data between this platform and our server-side. The result of data upload is a data reference that could be subsequently used in the service invocation (i.e. call- by-reference). In the client-side, files are also shown in a “Listview”, which has been improved with buttons to view, delete, and download them (see Fig. 2c). These files can also be referenced in the input parameters once the service execution interface has been generated. This is accomplished using a minified version of a “Listview” inside a modal menu.

## Results

This section presents the life cycle of the application and the browsing, composition, execution, and monitoring of a registered workflow. A software user manual is supplied in Additional file 2. The presented example and others are supplied in Additional file 3.

### Life cycle

**Fig. 4:**
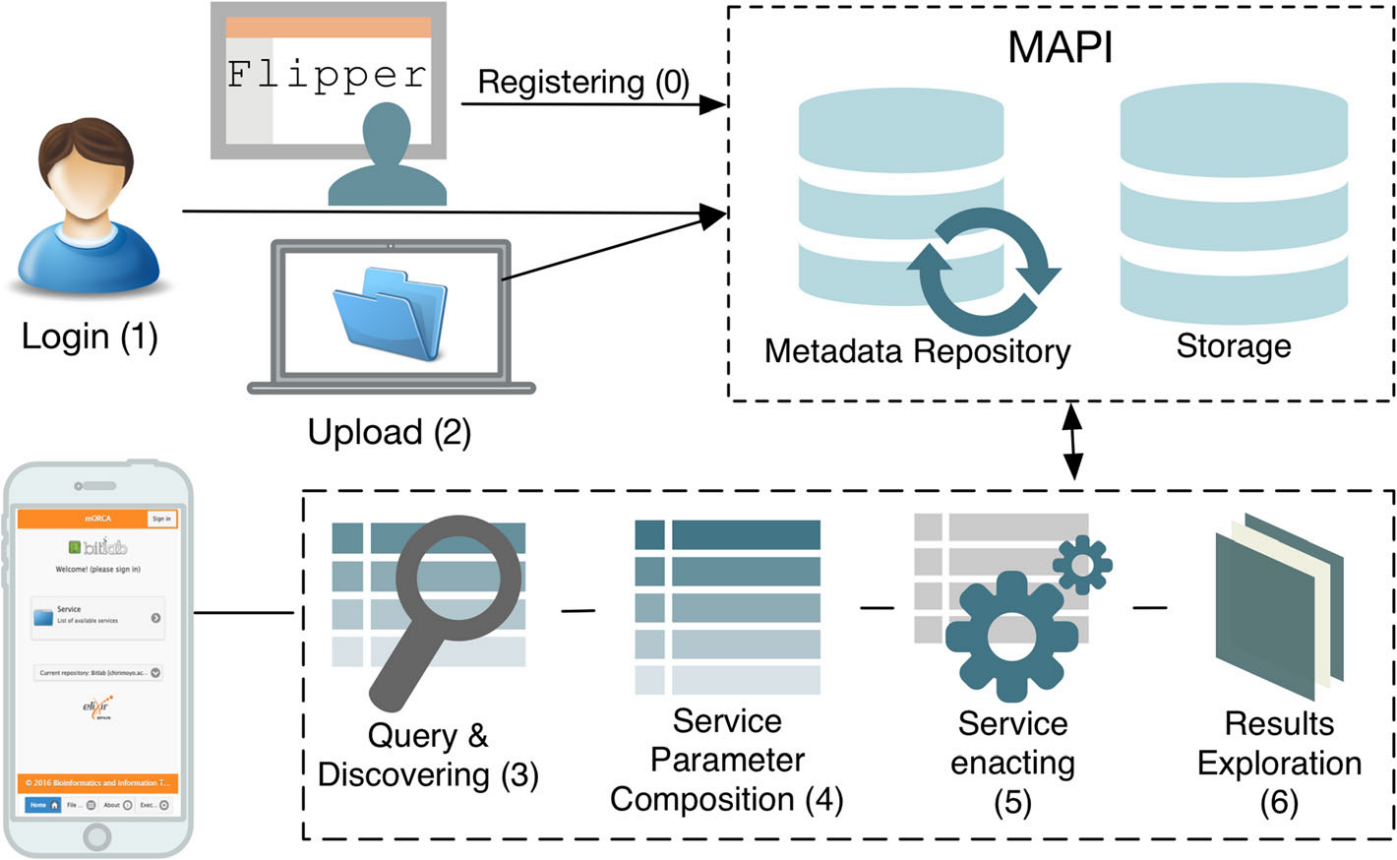
Life cycle for: (0) service registering; (1) authentication of the user in the system; (2) data file upload; (3) service browsing and discovering through the hierarchically tree; (4) filling of the dynamically generated service interface; (5) service execution from the client; and (6) results exploration. See main text for more details

Figure 4 illustrates the usual procedure for navigating, searching, invoking, and monitoring services from Web Service clients in external resources (such as regular servers, clusters, or even in the cloud environment). In general, the process consists of a series of stages. The prerequisite (0) is to store the metadata of the service(s) into the Web Services catalogue using the Flipper [31] tool (this is typically done by the repository administrator and is done a single time). At this point the service information is ready for use to the client. The first actions required from the end-user are to (1) authenticate in the system and (2) upload the data. The client then allows the user (3) to browse and discover the appropriate service, and once selected, the user needs to (4) fill out the service parameters including references to the data uploaded in step 2. At this point, the client lets the user (5) invoke the service, monitor it, and send the parameters to the MAPI execution module. As a final step, (6) the client allows the results to be explored once they become available.

### Diving into the mORCA application

The power of accessing Web Services via a mobile client is proved by presenting an exercise focused on the execution of a workflow composed of a diverse set of proprietary and third-party services. This workflow, which is registered in the BITLAB repository as ‘Homology Search And Phylogenetic Study’, uses a sequence ID to retrieve a sequence from the Uniprot database and produces a dendogram with the similar sequences.

This workflow is composed of the following proprietary and third-party services:

- Get amino acid sequence: Retrieves an amino acid sequence from the Uniprot database using the sequence ID.
- Run BLAST (EBI). This runs a BlastP homology search in the EBI server using the UnitprotKb database with the retrieved sequence. This is an external EBI service. The output is the BLAST report.
- Get best hits from Blast: This extracts the sequences most related to the query from the BLAST report using an E-value threshold. In this workflow, this value is set by default to 0.02 (the service itself can change this value). The output is a collection of sequences each with an ID and namespace.
- Get amino acid sequence collection: This service returns a set of amino acid sequences corresponding to the given ID/Namespace pairs. The output is a file with the retrieved sequences.
- Run ClustalW: The ClustalW algorithm is used to perform a multiple sequences alignment using the set of retrieved sequences. The output of this service is the sequence alignment reflecting sequence similarity between the analysed sequences.
- Run create tree from ClustalW: Finally, the relations obtained with the multiple alignment are used to build a dendrogram tree with Newick format.

Initially, the following steps must be performed (the full exercise is provided for demonstration purposes; see Additional file 3): (a) authenticate into the mobile client for privacy purposes; (b) choose the catalogue of services to be used (in this case the BITLAB catalogue); (c) discover the workflow by navigating or searching in the repository. Once the workflow is located, a swift query to the server-side is performed to retrieve the service metadata; (d) with the retrieved information the graphical parameters composition panel is dynamically built by the client; and (e) after filling the service parameters, the workflow can be executed. After invoking the workflow, the application enables execution status to be monitored. Finally, the results of the invocation and the intermediate files, which are stored in the server, can be consulted and retrieved for future use.

## Conclusions

Mobile devices are now of great relevance, not only because of their social impact and the way individuals use them to perform daily tasks, but also because of their strong potential influence on the scientific domain [32]. The ubiquitous, universal availability and other benefits of such devices have been emphasized along this document. However, there are still several use cases where mobile devices could make the difference, and should be also addressed, such as workflow generation and visualization, sharing capabilities using QR codes or Bluetooth, data representation, etc.

The transition from an application written for a WIMP desktop environment to a mobile device involves the loss of some characteristics of WIMP environment. The new possibilities offered by mobile devices neither exist nor are possible or needed in traditional applications. Among the most well-known functions is the hovering effect that provides the user with immediate information when the cursor hovers above an interactive item. This function is impossible in touch-based interactions. However, such effects as “pinching” (with a finger and thumb), rotating, two-finger sliding, and so on, are challenging but not impossible in WIMP interfaces. Some examples include multi-touch trackpads that can provide such effects within a desktop environment.

A further limitation is screen size, which restricts the quantity of information that may be displayed. However, screen size is not the only issue when designing interfaces for mobile applications. An additional issue is associated with the accuracy of the input devices (i.e. fingers in mobile devices) in comparison to the greater precision of mouse pointers. These limitations have been overcome by designing specific user interfaces with a responsive and clean design and with bigger components by which to introduce data.

Even though a variety of mobile devices (e.g.Android or Windows Phone) already have file managers, these devices remain unable to support large data file uploads, despite the user is using a Wi-Fi connection. Given the foregoing and other reasons, such as avoiding file re-transmission, we made the decision to decouple data upload from service invocation. This led to the development of several data transfer modules that use well-known data management tools such as Globus Online and Amazon S3. User authentication is needed to preserve privacy in data storage and service execution on the server-side.

The development tools inherited from the web development model using HTML5, JavaScript and other libraries are now mature enough to provide functional applications compatible with the majority of mobile operating systems (e.G. *ios*, Android and Windows Phone). The need of only writing once the application code significantly reduces the cost and time to get it running in all the available platforms. The main drawback is the limited access to the offered native capabilities. The hybrid programming model removes such limitation, enables the application distribution in the main Stores, and permits the offline working mode. These features motivated our change from the web to the hybrid programming model, being the latter the preferred choice to develop platform independent applications.

Repositories have been presented as an effective method for storing service metadata. The main issue relies on the dissemination and the lack of standardization of this metadata. Furthermore, the huge amount of available services makes the task of creating interfaces for all of them extremely difficult. Our system is another indicator of the pressing need for a common representation to facilitate the use of these metadata and, consequently, the catalogued services.

One of the problems related to the large data sets is the computation time needed to obtain results. For this reason, mobile devices are of interest regarding launching these kinds of services anytime, anywhere. To exploit their multitasking capabilities, these clients should implement non-blocking methods to avoid wasting time. To achieve this, we have implemented asynchronous methods combined with a monitoring middleware, which allow the user to keep in touch with execution status.

Future work should address which kind of visualization is appropriate in these devices given that screen size limits the information that can be represented. We suggest that preview methods linked to each data type should be implemented in order to add value to the obtained results. In addition, future work related to the possibility of storing the generated interfaces will lead to improvements in the smoothness of system transitions.

In conclusion, this article has described a mobile application, which is capable of browsing various service catalogues, dynamically generating service invocation interfaces depending on their metadata, executing the services and monitoring how such executions are progressing. The presented application is based on our initial prototype for mobile clients, which already was built following a thorough study of traditional Web Services clients for personal computers. Currently, mobile devices are also becoming important in the scientific world and we believe that they will be widely used in the near future. The application presented is a step forward to addressing such clients.

## Additional files

Additional file 1: State-of-the-art Apps. A PDF file with a full list of apps belonging to the stores of the main mobile platforms, its descriptions, links, and icons. (PDF 1546 kb)

Additional file 2: User Guide. A PDF file containing a user guide for the application, explaining the interface and the main operations. (PDF 2411 kb)

Additional file 3: Guided Exercise. The exercises described in the manuscript, step by step with screenshots and results. (PDF 2767 kb)

## Abbreviations

BIBM: Bioinformatics and Biomedicine
EBI: European Bioinformatics Institute
EMBL: European Molecular Biology Laboratory
GUI: Graphical User Interfaces
HCI: Human Computer Interaction
INB: Spanish Bioinformatics Institute
NCBI: National Center for Biotechnology Information
REST: Representational State Transfer
SOAP: Simple Object Access Protocol
UX: User Experience
WIMP: Windows, Icons, Menus and Pointers

## References

[1] EBI: EBI: European Bioinformatics Institute: www.ebi.ac.uk. 2016. www.ebi.ac.uk. Last accessed 24 Nov 2016.

[2] NCBI: NCBI: National Center for Biotechnology Information: http://www.ncbi.nlm.nih.gov/. 2016. http://www.ncbi.nlm.nih.gov/. Last accessed 24 Nov 2016.

[3] INB: INB: The Spanish Institute for Bioinformatics: http://www.inab.org/. 2016. http://www.inab.org/. Last accessed 24 Nov 2016.

[4] of Manchester, T.U.: NCBI: National Center for Biotechnology Information: http://bit.ly/15m2Rtp. 2011. http://bit.ly/15m2Rtp. Last accessed 24 Nov 2016.

[5] KK, JB. SimAlign Information: http://bit.ly/188GlGo. 2011. http://apple.co/2fsjTvr. Last accessed 24 Nov 2016.

[6] Varambhia, H.N. Oh BLAST it! Information: http://bit.ly/1yYS32q. 2013. http://bit.ly/1yYS32q. Last accessed 24 Nov 2016.

[7] Cheung, V., Heydekorn, J., Scott, S., Dachselt, R. Revisiting hovering: Interaction guides for interactive surfaces. In: Proceedings of the 2012 ACM International Conference on Interactive Tabletops and Surfaces. ITS ‘12, pp. 355–358. ACM, New York, NY, USA (2012). doi:10.1145/2396636.2396699. http://doi.acm.org/10.1145/2396636.2396699

[8] Deegan, R. Managing distractions in complex settings. In: Proceedings of the 15th International Conference on Human-computer Interaction with Mobile Devices and Services. MobileHCI ‘13, pp. 147–150. ACM, New York, NY, USA (2013). doi:10.1145/2493190.2493228. http://doi.acm.org/10.1145/2493190.2493228

[9] Plumlee MD, Ware C (2006). Zooming versus multiple window interfaces: cognitive costs of visual comparisons. ACM Trans Comput-Hum Interact.

[10] Pfeifer Vardoulakis, L., Karlson, A., Morris, D., Smith, G., Gatewood, J., Tan, D.: Using mobile phones to present medical information to hospital patients. In: Proceedings of the SIGCHI Conference on Human Factors in Computing Systems. CHI ‘12, pp. 1411–1420. ACM, New York, NY, USA (2012). doi:10.1145/2207676.2208601. http://doi.acm.org/10.1145/2207676.2208601

[11] Acharya, C., Thimbleby, H., Oladimeji, P. Human computer interaction and medical devices. In: Proceedings of the 24th BCS Interaction Specialist Group Conference. BCS ‘10, pp. 168–176. British Computer Society, Swinton, UK, UK (2010). http://dl.acm.org/citation.cfm?id=2146303.2146329

[12] Insight, C. Smartphone Sales to Peak in Western Markets in 2017 as They Enter New Phase of Maturity Information. 2015. http://bit.ly/1NoQIVq. Last accessed 24 Nov 2016.

[13] Andrade, P.R.M., Albuquerque, A., Frota, O.F., Silveira, R.V., da Silva, F.A.: Cross platform app: a comparative study. CoRR abs/1503.03511. 2015.

[14] Ison J, Rapacki K, Ménager H, Kalaš M, Rydza E, Chmura P, Anthon C, Beard N, Berka K, Bolser D, et al. Tools and data services registry: a community effort to document bioinformatics resources. Nucleic Acids Res. 2015:1116. 10.1093/nar/gkv1116 PMC4702812 26538599

[15] Karlsson J, Trelles O, Freitas AT, Navarro A (2012). jORCA and Magallanes sailing together towards integration of web services.

[16] Ramirez S, Munoz-Merida A, Karlsson J, Garcia M, Perez-Pulido AJ, Claros MG, Trelles O (2010). MOWServ: a web client for integration of bioinformatic resources. Nucleic Acids Res.

[17] Oinn T, Addis M, Ferris J, Marvin D, Senger M, Greenwood M, Carver T, Glover K, Pocock MR, Wipat A, Li P (2004). Taverna: a tool for the composition and enactment of bioinformatics workflows. Bioinformatics.

[18] Gordon PM, Sensen CW (2007). Seahawk: moving beyond HTML in web-based bioinformatics analysis. BMC Bioinformatics.

[19] Li W, Cowley A, Uludag M, Gur T, McWilliam H, Squizzato S, Park YM, Buso N, Lopez R (2015). The EMBL-EBI bioinformatics web and programmatic tools framework. Nucleic Acids Res.

[20] Diaz-del-Pino S, Karlsson TJM, Falgueras Cano J, Trelles O. In: Ortuño F, Rojas I (eds.) Mobile Access to On-line Analytic Bioinformatics Tools. Springer, Cham; 2015. pp. 555–565. doi:10.1007/978-3-319-16480-953, 10.1007/978-3-319-16480-953

[21] Karlsson J, Trelles O (2013). MAPI: a software framework for distributed biomedical applications. J Biomed Semantics.

[22] Wilkinson MD, Links M (2002). BioMOBY: an open source biological web services proposal. Brief Bioinform.

[23] jQuery Project, T.: jQuery Mobile. 2010. http://jquerymobile.com. Last accessed 24 Nov 2016.

[24] jQuery Project, T.: jQuery. 2010. http://jquery.com

[25] Apache Cordova. https://cordova.apache.org/. Accessed 18 Dec 2017.

[26] Dahl, R.L.: NodeJS. 2009. http://nodejs.org. Last accessed 24 Nov 2016.

[27] Inc., M.: MongoDB. 2009. http://www.mongodb.org/. Last accessed 24 Nov 2016.

[28] Rios J, Karlsson J, Trelles O (2009). Magallanes: a web services discovery and automatic workflow composition tool. BMC Bioinformatics.

[29] Globus: Globus Online. 2013. https://www.globusonline.org. Last accessed 24 Nov 2016.

[30] Amazon: Amazon S3. 2006. https://aws.amazon.com/s3. Last accessed 24 Nov 2016.

[31] Torreno, Oscar and Trelles, Oswaldo: Easily Registering Bioinformatics Services Metadata. 2014.

[32] Nature: The scientist and the smartphone. Last accessed 24th of November 2016. 2010. http://go.nature.com/2gdnq2T

