# Supplementary material for: mORCA: ubiquitous access to life science web services
Source: BMC Genomics. 2018 Jan 16;19:56. doi: 10.1186/s12864-018-4439-x (PMC5771032; doi:10.1186/s12864-018-4439-x)
Supplement: Supplementary file 1 — State-of-the-art Apps. A PDF file with a full list of apps belonging to the stores of the main mobile platforms, its descriptions, links, and icons. (PDF 1546 kb) [file 12864_2018_4439_MOESM1_ESM.pdf]

mORCA: Ubiquitous access to life science Web Services

Sergio Diaz-del-Pino[1], Oswaldo Trelles[1] and Juan Falgueras[2,*]

{sergiodiazdp, ortrelles, juanfc}@uma.es

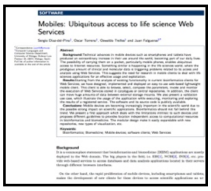
SOFTWARE

Mobiles: Ubiquitous access to life science Web Services

(1) Computer Architecture Department
(2) Computer Languages and Computer Science Department
University of Malaga, Boulevard Luis Pasteur 35, 29071 Malaga, Spain
(*) Corresponding author

Document submitted to 'BMC Genetics'; in review process.

---

mORCA

State-of-the-art apps

http://chirimoyo.ac.uma.es/morca/app

Contact: sergiodiazdp@uma.es (Sergio)
ortrelles@uma.es (Oswaldo)
juanfc@uma.es (Juan)

Diciembre 2017

Bioinformatic available mobile apps 2016-07

Category*	App name	Performs	Advantajes	Dissatvantajes	Link	
2	CloningBench	Helps to do cloning experiments, in every detail based on tables for each kind of enzimes, etc.	Up to the point, it helps with the details for this kind of experiments. Free	Quite specific	https://itunes.apple.com/es/app/cloningbench-by-invitrogen/id458617777?mt=8	
2	Promega	View, run and annotate Promega protocols. Run timers directly from the relevant steps, and when complete, email the annotated protocols for printing, etc	Interesting tool for this lab users. Free	Specific	https://itunes.apple.com/es/app/promega/id307546949?mt=8	
1	Phylogram	Visualization of phylogenetic tree	Phylogram allows visualisation of the phylogenetic trees resulting from bioinformatics sequence analyses	Just for iPhone, not intuitive, does not hanlde landscape, can't get to file selection. Low interactivity. Difficult to zoom-handle-intertact even with small phylogenetic trees.	https://itunes.apple.com/us/app/phylogram/id399814043?mt=8&ls=1	
1	Biocatalogue	Browse the catalogue for services, service providers. REST endpoints and SOAP Operations	Informational app, handles landscape and portrait. Free	Is more like a web site. Information is minimal, no links. Unorganized, plain lists, non hierarchical nor contents organized. Quite difficult to browse or search through.	https://itunes.apple.com/us/app/biocatalogue/id450120348?mt=8&ls=1	
2	EdgeBio	It allows access to all content from the EdgeBio blog, website and their online communities	Unifies all digital resources of EdgeBio blog. Free.Interacts via scial networks	It is more a link to videos and on line resources than a tool or app. Not access from all countries	https://itunes.apple.com/us/app/edgebio/id515313935?mt=8&ls=1	
1	SimAlign	iOS touch application that interfaces with NCBI BLAST service to align a provided sequence or Accession.	Saves all queries allowing users to recall and resubmit searches. iOS and Android	It is not confortable to use, many dialogs for the only task it can do: NCBI blast. It depends on registered users. It is unable to handle files, you must copy-paste or type the entire sequence in a small textbox. It is based on registering and not free accounts.	https://itunes.apple.com/us/app/simalign/id432818873?mt=8	
1	SimGene	Interfaces with Simbiot, Ensembl, NCBI, etc, to retrieve annotation info for over 30-species, based on gene and symbol search	iOS and Android	Same user interface and uncomfortable handling as SimAlign. It is based on registering and not free accounts.	https://itunes.apple.com/us/app/simgene/id427772349?mt=8	
1	Oh BLAST It!	Executes BLAST	Simple. Free	Only Android. It doesn't have a real graphical user interface, but a kind of black screen where everything is displayed and entering as command lines	https://play.google.com/store/apps/details?id=com.bioinformaticsapp	
1	Gene Aligner	This program uses the Needleman-Wunsch and Smith-Waterman algorithms for pairwise gene global and local alignment, respectively.	Simple. Free. The sequences can be entered in two different ways: string of characters or NCBI accession number. The program can also generate a dot plot for the alignment.	Only Android. It doesn't have a real graphical user interface, but a kind of black screen where everything is displayed and entering as command lines	https://play.google.com/store/apps/details?id=itd_gene.activities	
2	NEB Tools /New England Biolabs Inc	Enzyme finder for restriction enzyme finding information on any NEB enzyme	Complete, well documented and organised. Free	Quite specific for this labs	https://itunes.apple.com/es/app/neb-tools/id350346827?mt=8	
3	RCSB PDB	Official mobile app of the RCSB PDB (Protein data bank)	Amazing database, with molecular 3D rendering.	Depends on internet connection. Only querying	https://itunes.apple.com/us/app/rcsb-protein-data-bank/id529153183?ls=1&mt=8	
2	PCR Essentials	Interact Remotely with your ProFlex™ PCR system. product information on Taq Polymerases, dNTP, Gels and stains, RT-PCR products, cDNA products and master mixes, etc.	Smart interface, high quality app. Free	Specific	https://itunes.apple.com/es/app/pcr-essentials/id606990640?mt=8	
3	DrugBankWP	Chemical, pharmacological and pharmaceutical database	Complete	WindowsPhone only	http://www.windowsphone.com/es-es/store/app/drugbankwp/968f1dbe-0e67-4c2a-b733-16bfcfd4f51c	
1	DNAApp	Open ab1 files	Open and visualise sequences from Dropbox (in iOS). New versions save also in fasta format	Simple. Not well adapted to common gestures in mobiles.	https://itunes.apple.com/us/app/dnaapp/id854944694?mt=8	
4	Evolutionary Biology	Concept to teach evolutionary concepts	Simple	More as web site than appication. Simple. It covers only quite a few topics in Bioinformatics, it's very simple and take no advantage of the interactive capabilities of the device. Not free	https://itunes.apple.com/us/app/evolutionary-biology/id513464425?mt=8&ls=	
4	Gen Tutor	iOS student app for students taking a standard College level course in Genetics	Extensive and complete based on levels of knowledge. Free	A tool for students, following a course	https://itunes.apple.com/es/app/gene-tutor/id534062019?mt=8	
4	Gene Link	Quick short course in molecular genetics	High quality material. Wide range and well organized number of topics. Tools for researchers. Free	Only tools for handling one sequence, simple ops	https://itunes.apple.com/es/app/array-genetic-tools-from-gene/id399712676?mt=8	
4	Nature ENCODE	It helps to navigate through the 30 papers published in Nature, Genome Research and Genome Biology as the ENCODE Project	Amazing presentation of the state of the art in the most important papers published	iPad only	https://itunes.apple.com/es/app/nature-encode/id553487333?mt=8	
4	MyGenome/ Illumina, Inc	Explore real human genome	Graphic, health implications, educational, quite complete. Good graphics, a lot of information on genetic deseases. Cheap.	iPad only	https://itunes.apple.com/es/app/mygenome/id516405838?mt=8	
4	Human Genome	Obtain graphical info between diseases and genes, syndromes or traits	Graphical, pedagogic, free	iPad only	https://itunes.apple.com/es/app/human-genome/id576939342?mt=8	
2,4	MIQE qPCR	Helps with technical support on particular qPCR experiments. Based on characteristics, it offers articles and depending on your problem, particular advise	Essentially useful. Kept updated, with active version supporting. Free	Quite specific, documentative.	https://itunes.apple.com/es/app/miqe-qpcr/id423650002?mt=8	
2	Real-time PCR	Clearly organised set of documentative resource on experiment preparation and results interpretation on PCR	Attractive, easy to use, a lot of information. Update. Free	Specific, documentative	https://itunes.apple.com/es/app/real-time-pcr/id523157743?mt=8	
3	Talking Glossary of Genetics	By National Human Genome Research Institute, more than 250 common genetic terms pronounced and explained in an easy-to-understand way by leading scientists and professionals at the National Human Genome Research Institute (NHGRI).	Great pictures, explanations, comprehensive and balanced explanations on every genetic topic. Free	Heavy, only educational	https://itunes.apple.com/es/app/talking-glossary-of-genetics/id428340581?mt=8	
3	Protocolpedia	For lab researchers. Offline access to hundreds of complete protocols	Light, complete. Well organised and supported by real users. Includes videos. Free	Not updated since 2015	https://itunes.apple.com/es/app/protocolpedia/id396334248?mt=8	
3	BioTechLCD	Access to bioinformatics and genomic information	Free	More than an app is an informative tool-a blog	http://www.androidpit.de/de/android/market/apps/app/com.globetech.biotechdaily/biotech-daily	
3	BioGene	A search and information tool for biological search. A quick reference for any gene	Really documentative. Well organised. Free	Specific for genes	https://itunes.apple.com/app/biogene/id333180084?mt=8	
3,1	BioGenie	Multitask system to be used by biologist, scientist researchers, professors and students	Help using those services	Specific	http://bio-genie.com/node?page=2	
4	Gene Lab	iOS educative game on creating creatures based on their genes	It permits to experiment essentially with the Mendel laws and mutation concept	Simple concepts, only recreational	https://itunes.apple.com/es/app/gene-lab/id320762232?mt=8	
4	Gene Pool	Game. iOS	Amusement	Simple concepts	https://itunes.apple.com/es/app/gene-pool/id330408920?mt=8	

*** Categories**
1: Independent for directly dealing with specific bioinformatic problems
2: Depends on particular companies to access their software
3: Accesses and browses specific databases
4: Educational walkthrough and/or game to explore and learn bioinformatics

2. Comparison table

	Multiplatform	No. Different services	Property/Free	File management
**mOrca**	Yes	No limits	Free	Local/Remote
**Phylogram**	No	1	Free	Local
**Biocatalogue**	Yes	0	Free	Remote
**SimAlign**	Yes	1	Free	Local/Remote
**SimGene**	Yes	6	Free	Remote
**Oh BLAST It!**	No	1	Free	Local
**Gene Aligner**	No	1	Free	Local
**DNAApp**	No	5	Free	Local/Remote

This table includes the differences between some of the most relevant apps in the stores and their main functionalities. Being multiplatform is a double-edge characteristic because, as described in the manuscript, native apps are usually faster but restricted to one platform while hybrid apps are usually slower but can be executed in several platforms. The mORCA's main strenght is to be capable of execute a open number of services while others are specifically designed to run an specific software or a small amount of them. Furthermore, local or cloud file management is also an important feature in mobile devices due to capacity limits.

Legend

- Multiplatform: It can be executed in, at least, two different platforms (Android, iOS, Windows Phone, Linux…)

- No.different services: that can be executed from the application. Each service provides different functionality (i.e. Blast, clustal, etc.)

- File management: Local or remote file saving/reading
