# Supplementary material for: mORCA: ubiquitous access to life science web services
Source: BMC Genomics. 2018 Jan 16;19:56. doi: 10.1186/s12864-018-4439-x (PMC5771032; doi:10.1186/s12864-018-4439-x)
Supplement: Supplementary file 2 — User Guide. A PDF file containing a user guide for the application, explaining the interface and the main operations. (PDF 2411 kb) [file 12864_2018_4439_MOESM2_ESM.pdf]

Mobiles: Ubiquitous access to life science Web Services

Sergio Diaz-del-Pino[1], Oswaldo Trelles[1] and Juan Falgueras[2,*]

{sergiodiazdp, ortrelles, juanfc}@uma.es

(1) Computer Architecture Department
(2) Computer Languages and Computer Science Department
University of Malaga, Boulevard Luis Pasteur 35, 29071 Malaga, Spain
(*) Corresponding author

Document submitted to 'BMC Bioinformatics'; in review process.

---

mORCA: User Guide

This document is the *User Guide* of the application mORCA for mobile devices access to life science Web services.

http://chirimoyo.ac.uma.es/morca

Content

1 Getting started
  1.1 The main screen
2 Signing and registering
3 Services repositories
  3.1 Filtering text-box
4 Executing a service
  4.1 Using existing files as input for services
5 Regarding processes Monitoring
6 Upload and download files
7 File Browsing
8 References

Contact: sergiodiazdp@uma.es (Sergio)

November 2016

1. Getting started

Application access: mORCA is available in the App store by Apple and Play Store by Google. It is also available as web-app in the URL: http://chirimoyo.ac.uma.es/morca/app/

This application can be executed from a desktop web browser although is specially designed for mobiles devices. It has been tested and is compatible with the more common Internet browser: Internet Explorer +9, Firefox +30, Chrome +36, Safari +8 and Opera +12. Also through is +8 and Android +4.2.

1.1 The main screen

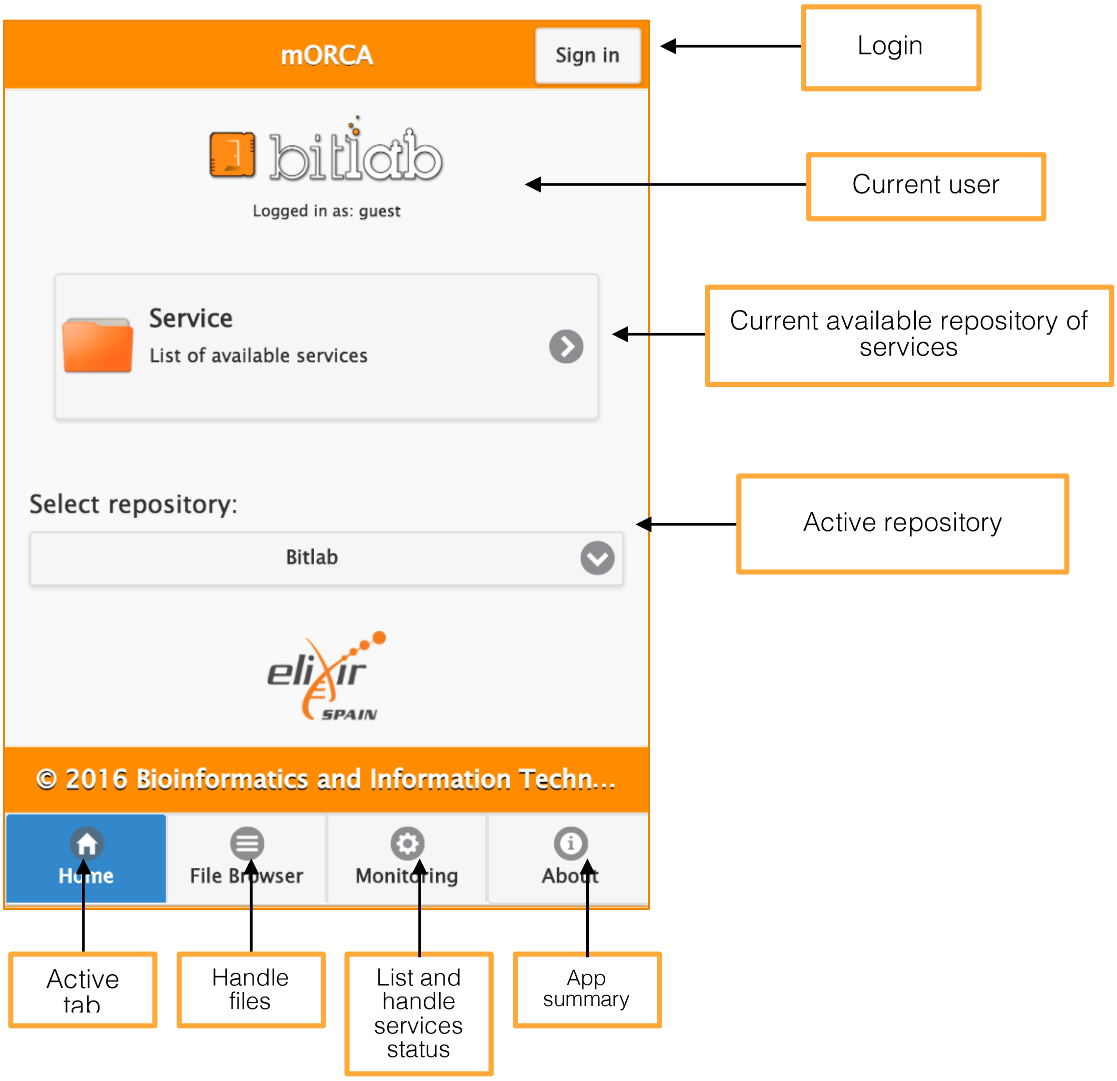

2. Signing and registering

Signing is mandatory if you want to keep your data in a private accont and It is previous to any other step. The button is available at the upper right corner of the "home" screen.

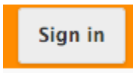

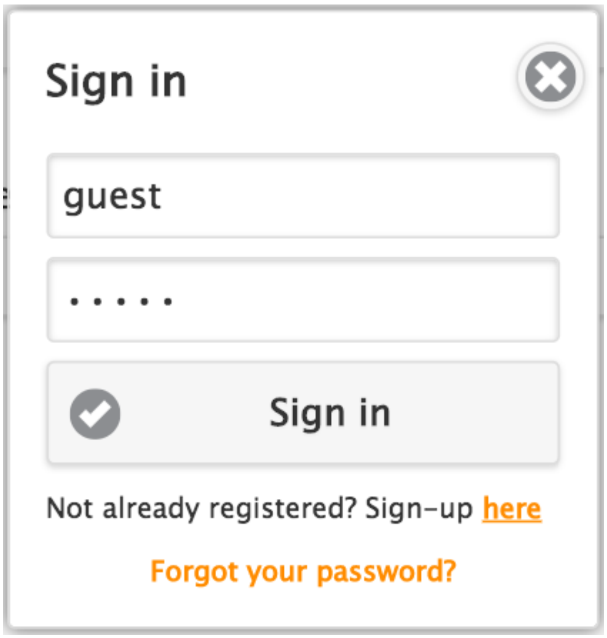

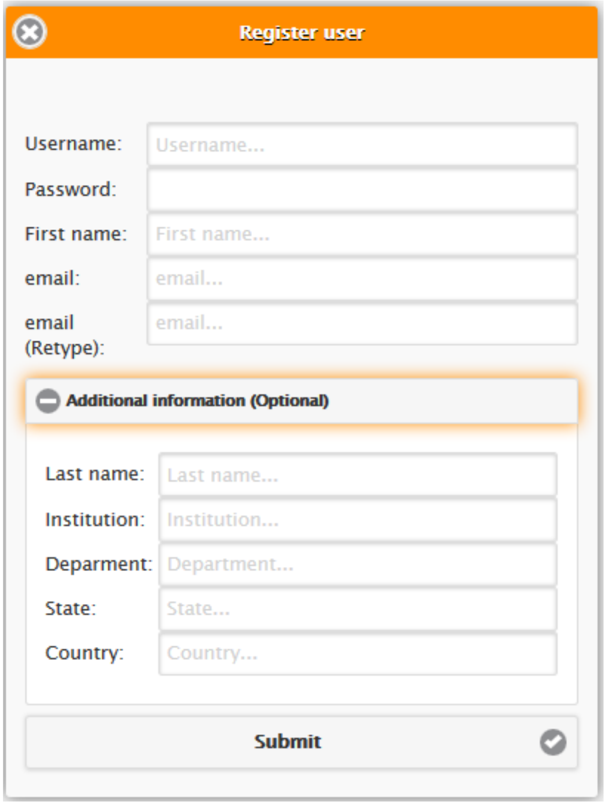

After signing the name of the user would appear at the top of the dialog. The user can logout and close access to their repository at any moment.

It is also possible to use a default guest/guest signing that gives you access to all mORCA functionality. However this "guest" credential can be shared by different users, therefore confidential data should no use this account.

Log-out button replace the "Sign-in" button for registered users. Log-out for guest users is automatically performed when change the user or abandon the system

To get a personal account you must register into the system, giving the opportunity of preserving privacy and personal cloud storage account for results.

New users: Registering procedure

To get credentials as new user a minimal set of information is requested. Additional information is highly recommended to also be completed

Keep in mind that even when exist a generic guest user, files in that directory are shared by all the users using such credential; and more important, that files are removed every two days

Forgot your password?

Please send an email to the system administrator: sergiodiazdp@uma.es or to ortrelles@uma.es with your user name. A new password will be sent to your registered email

3. Services repository

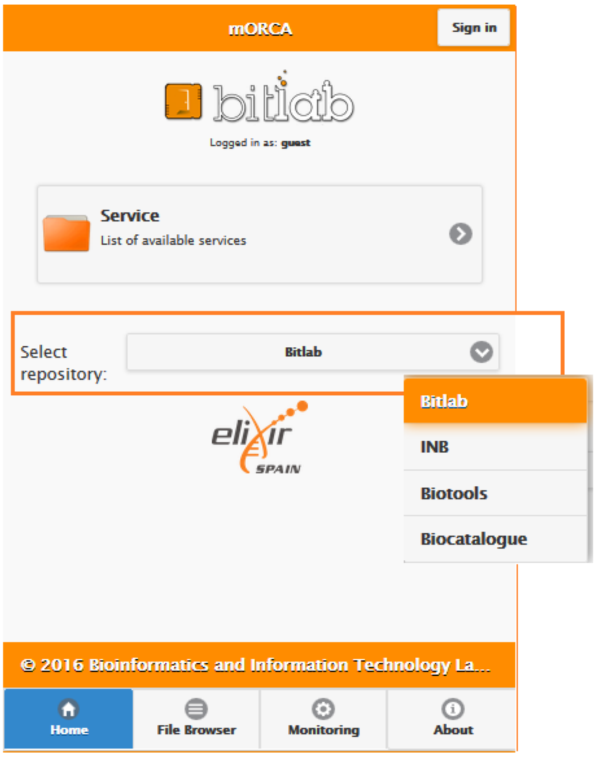

mORCA server-side is able to work with multiple repositories by implementing different accesses for each of the configured catalogues, decoupling the client-side from this task. BITLAB repository is used by default.

New repositories can be easily incorporated by written a small piece of software named "access" whose function is to map the data structure of the new repo into the data structure used by mORCA (MAPI).
(See Annex 1 for implementation details)

New services can also be incorporated to existing repositories. Flipper software (1) is used for this task. New services can be added by registered user or (preferably) by the system administrator for a better control of service quality.
(1) Torreno, O. & Trelles, O. (2014) easily registering bioinformatics services metadata. F1000Research 5:1787.

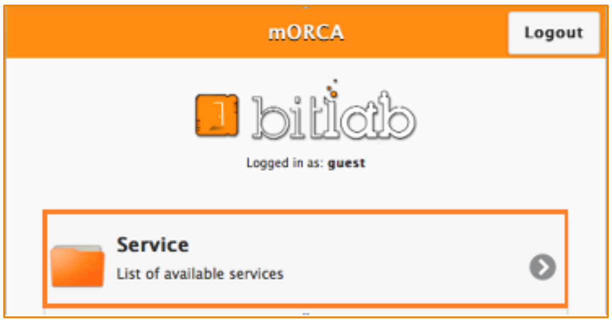

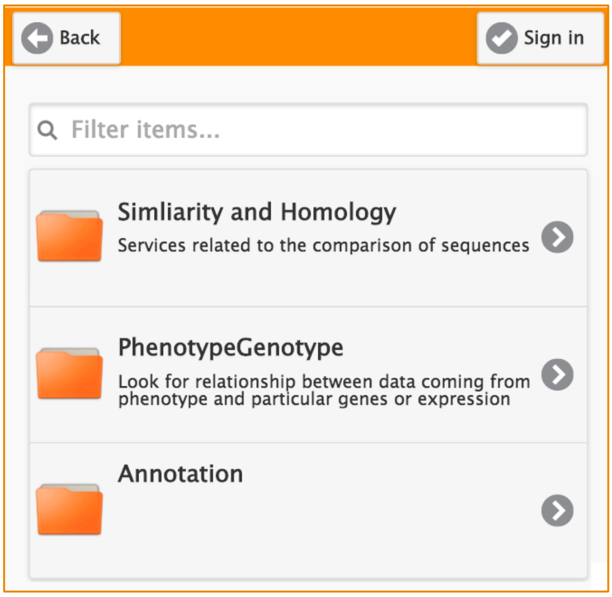

4. Browsing the catalogue

Once in the system as guest or as individual user, you can browse the list of services.

The home window contains a "Service" button to start browsing the catalogue content.

Services are organized in functional categories hierarchically organized in folders to facilitate browsing. You can choose one of these categories or previously filter dynamically this list to see only the categories of interest (see below: Filtering). Use the traditional style for moving through the different categories and services.

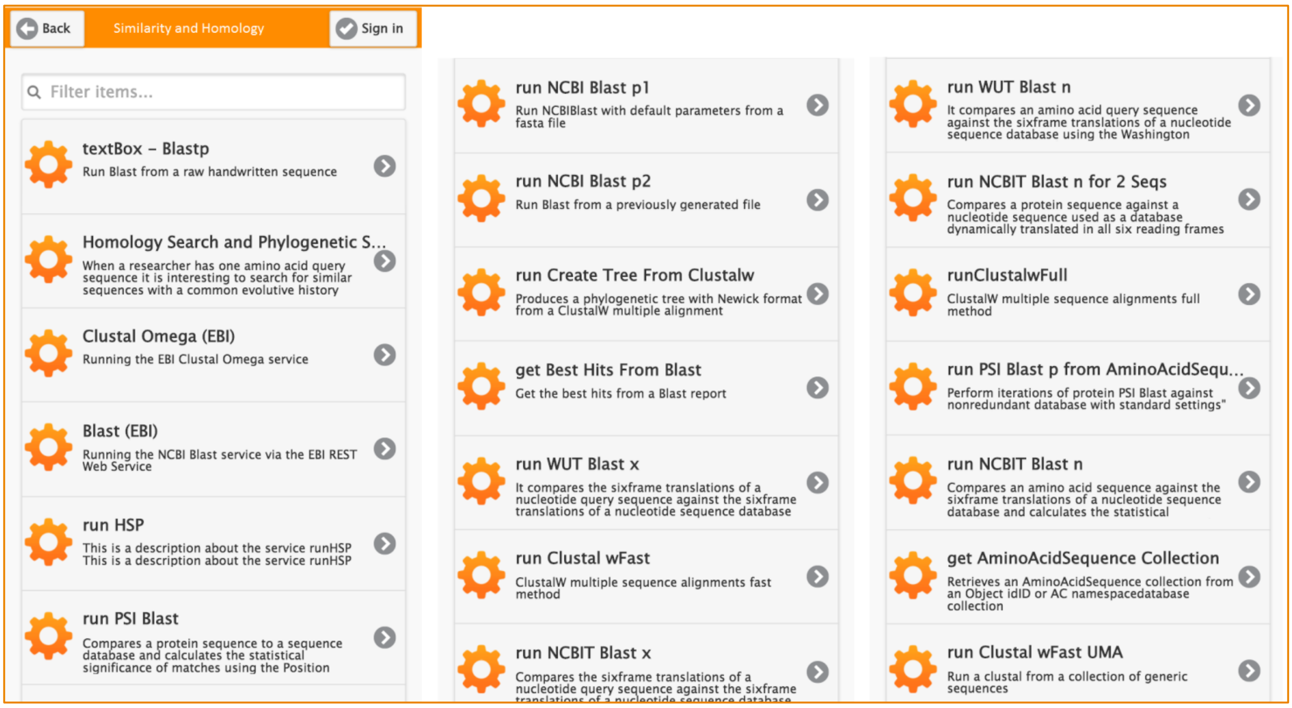

Touching this category will take us into a list of final services. In this case "**Similarity and Homology**" is the title the next offered list. For the selected category, the list of services is displayed. Depending on the number of available services in each category it would be necessary to scroll the page.

The "category description" is available in the page header.

This list of services display the name of the services and a short description under the titles with longer explanations for you better identify the specific goals of each one. These metadata are obtained from the service's repository and were set-up there during the registration procedure

3.1 Filtering text-box

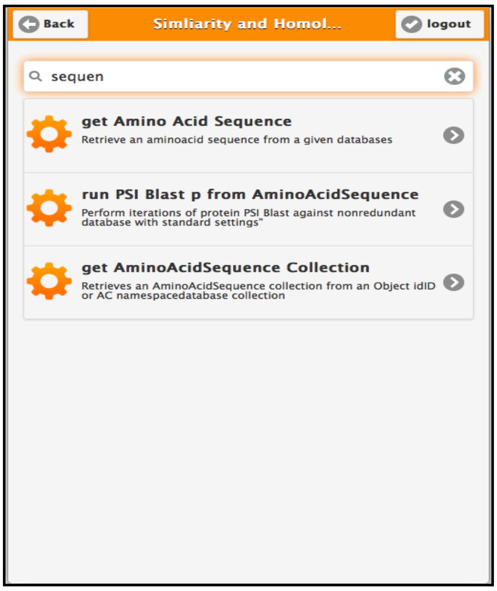

As the services list can be too long, filtering it with a dynamic search box as the one offered previously is yet more necessary.

Searching for the desired service can be done scrolling and browsing the list or simply typing its name into the heading dynamic filter text box offered. The searching algorithm implements fuzzy strategies.

In the figure, you can see only the services related to sequences handling are listed then giving you a quick glance of what we have available.

4. Executing a service

A service is invoked when the user touch / click on it. A new window exposing the service's parameters is displayed. In general, computational services require parameters to customize their behavior. Parameters can be of different flavor; from existing input files to simple numeric or text values. These parameters depends on the services, therefore here we describe them in a general context.

Some previous comments are necessary regarding input / output data files. In general mobile devices are still not prepared to deal with large data files; therefore we opted by decoupling the data transfer from the service invocation in an attempt to ease the task and to avoid file re-transmission.

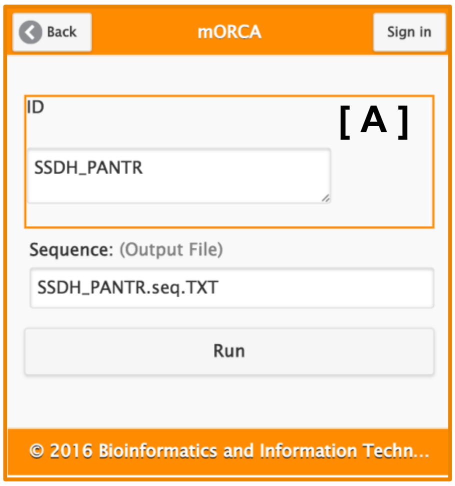

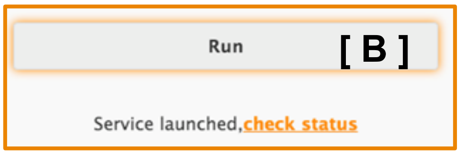

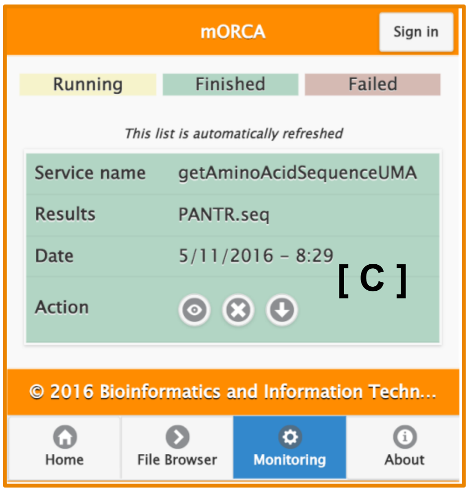

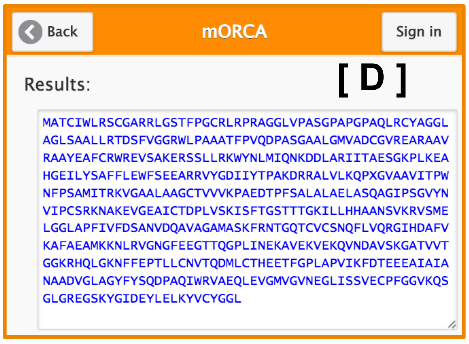

It is also possible that a service is executed in pipeline mode, using as input, the output files from another service previously executed. In this situation the file will already be in the file user space.

For these reason, some input files must be already available in the system. To do it, an upload procedure is available (see below).

For instance, let's suppose we start retrieving a sequence using the getAminoAcidsequence service. In this case **[A]**, we use SSDH_PANTR as sequence ID and specify SSDH_PANTR.seq.TXT as output file. In this simple case we only need to complete two names in text-boxes

The service is executed **[B]** and result appear in the user file system (in this case the image **[C]** represents the monitoring dialog screen), from which is also possible to visualize the content of this text file **[D].**

4.1 Using existing files as input for services

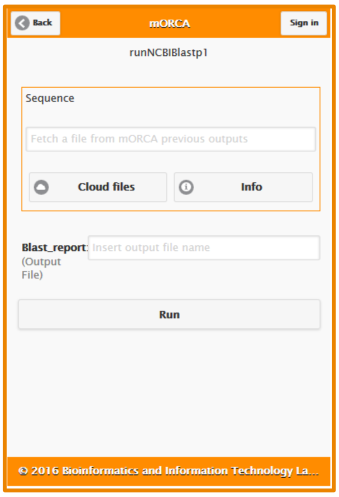

Now, we want to execute a "run NCBI Blast p1" service (available in the similarity and homology folder). This service uses a query sequence to search for similar sequences in a protein database).

In this case the service request an input file (the query sequence). (Note: other parameters have been omitted for the sake of simplicity)

In this case, just below the Sequence input box, appears the "Cloud files" button.

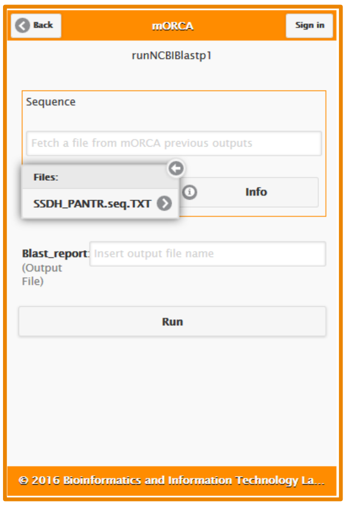

Clicking it, a pop-up menu displays with the list of available files in the user files system. In this case, we have only executed the get Amino Acid Sequence and have SSDH_PANTR.seq.TXT file (a protein sequence file).

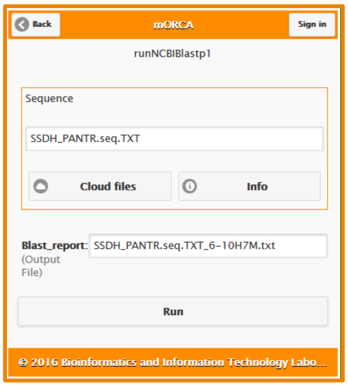

Click over the SSDH_PANTR.seq.TXT name, the file name incorporates as input file; and automatically a default file name for the output is proposed and is formed with the same name as the input file, plus day + hour. The user can change its file name.

4.2 Other parameter

Services display a number of different parameters. Since the application build-ups interfaces on the fly, here we present –in general—these different type or parameter, in this case using an example.

Depending on the way the service was registered some of these parameters can have a default values or not, can be endowed with a text readable information that can be displayed as additional information. This is an important reason to call the attention in the registering procedure.

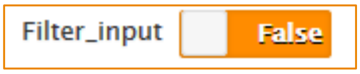

On-Off defined as Boolean during registering

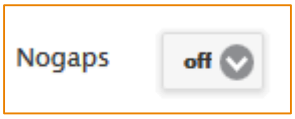

On-Off as an string with allowed values for this parameters (in this case only two values, On and Off)

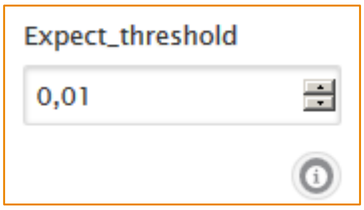

Float/Integer values as a numeric text box input.

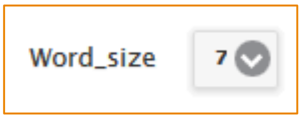

Numeric field with predefined values

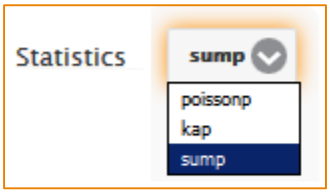

Alphanumeric string with predefined values

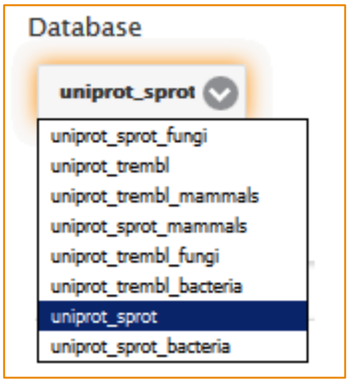

Another example (as a List) of alphanumeric predefined values

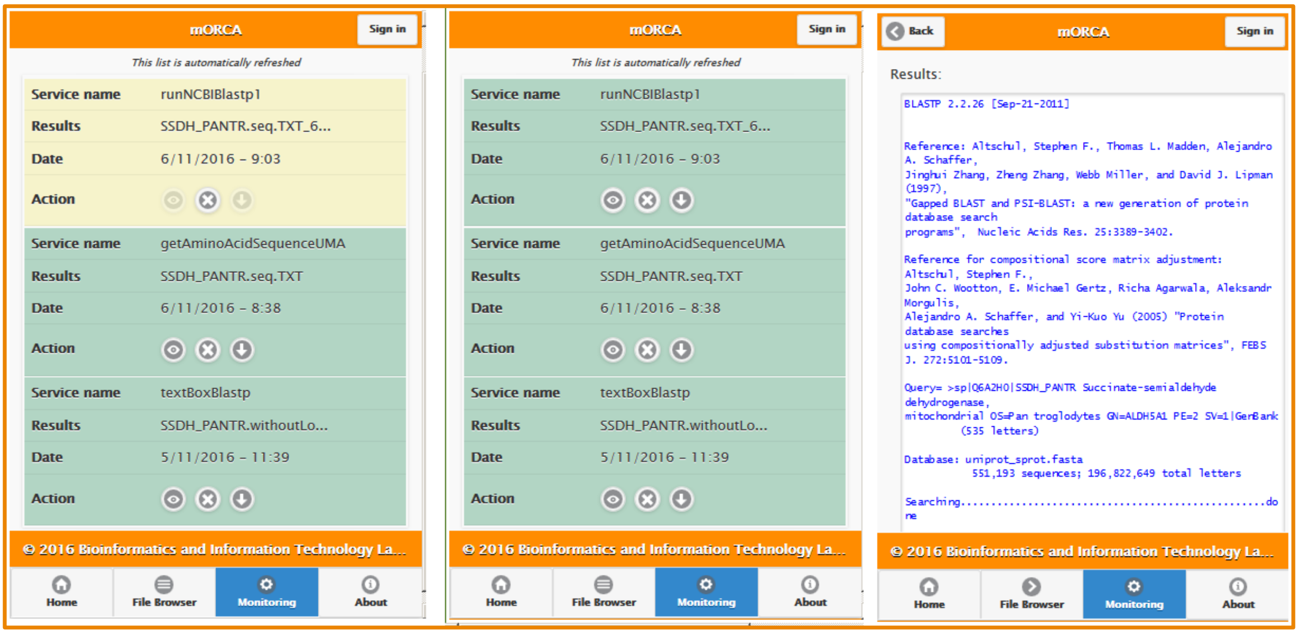

Finally, user can monitor the execution. On the left image, the process is still “running”; in the middle, the process has finished and shows the results that can visualized (right hand side of the image composition).

5. Regarding processes Monitoring

mORCA launches services in asynchronous mode; therefore, several services can be active at a given moment. The monitoring section allows to follow-up the status of services, using a color nomenclature:

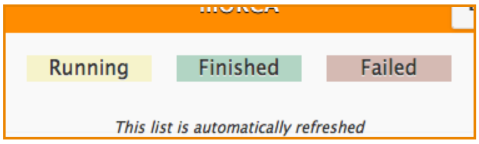

(See monitoring section) Running, finished, and failed processes are displayed in yellow, green and brown respectively.

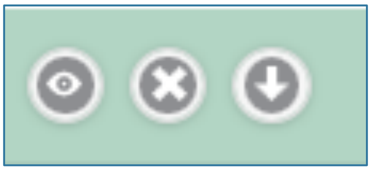

These icons express the available options to manipulate files. The first on the left is for visualization. We plan to associate each type of “file extension” to a viewer; the middle icon is used to delete a file and finally the icon on the right is for file download

Noteworthy observe, download have sense in such devices with capacity to store the file, which is normally available in mobile devices. Since this application can also be invoked from a desktop computer, the file will be stored in the local file system

6. Upload and download files

Download files

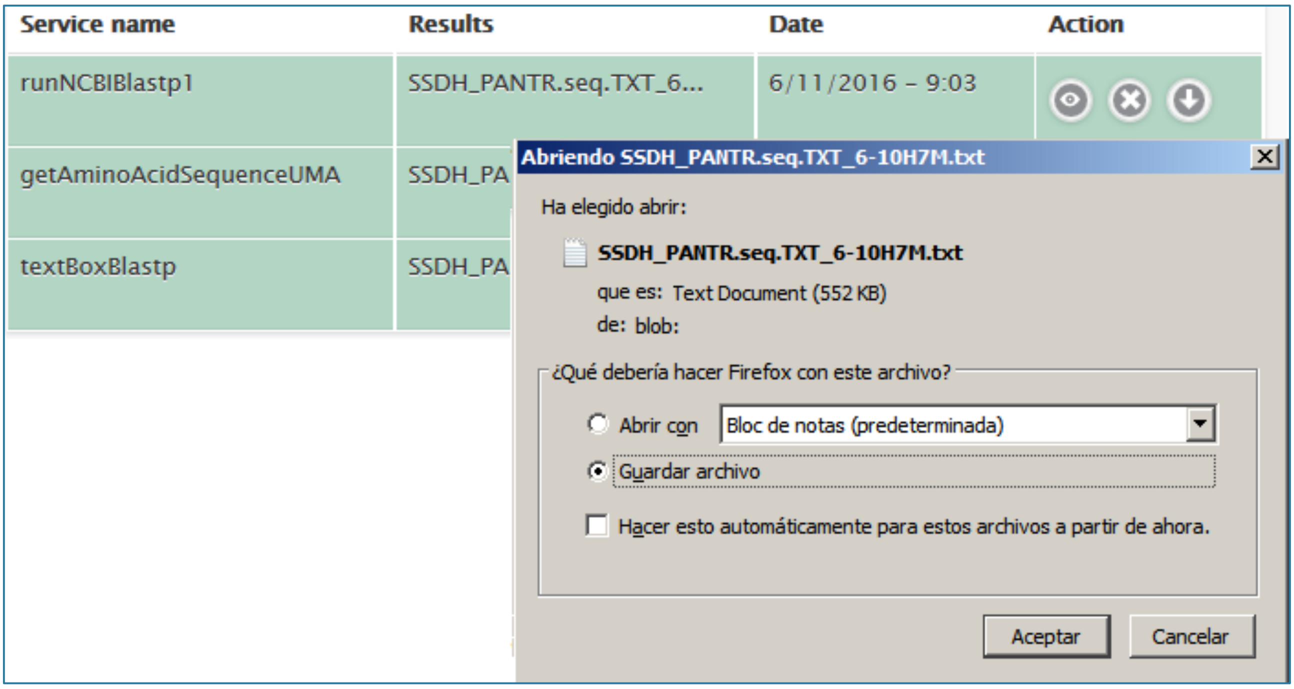

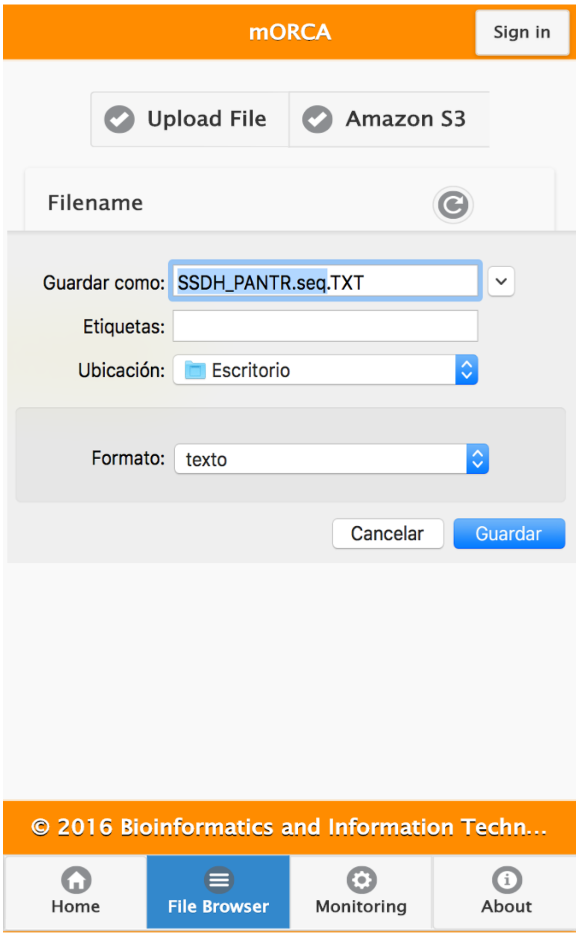

Files can be downloaded and uploaded to the system through the interface. The download is allowed throught all the devices able to handle files as Android or Windows.

The upload is allowed for most devices through the native filesystem or other cloud storage as Dropbox, AmazonS3, etc. This upload is recommended only for small files.

In the images, the download interface (Firefox under Windows and Chrome browser under MacOS)

Upload files

Two options are provided in order to upload files from the mobile device to the cloud. From the 'File browser' tab, the 'Upload file' button allows the user to upload files stored in their local file system (always that the device allows the access to it, like most of the Android devices) or from other clouds storages installed in the device. In the screenshot the menu view for a iPhone 6 with iOS 10 using Dropbox and Google Drive.

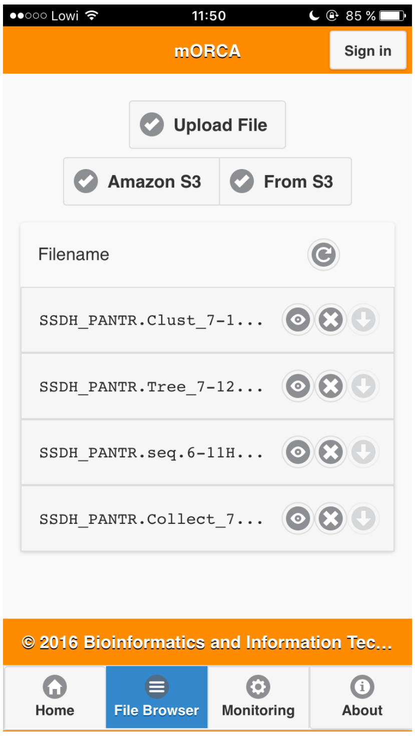

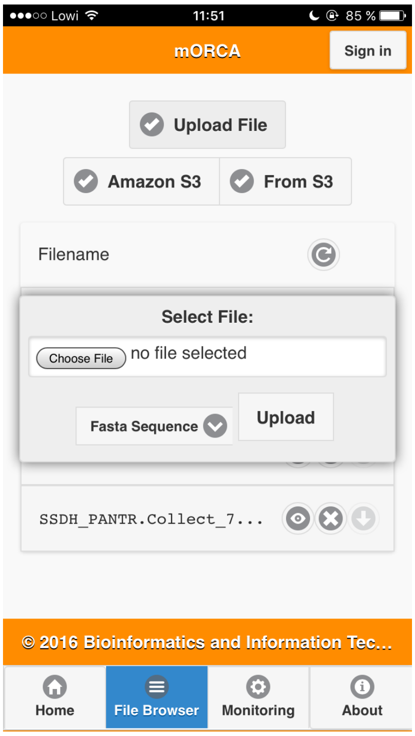

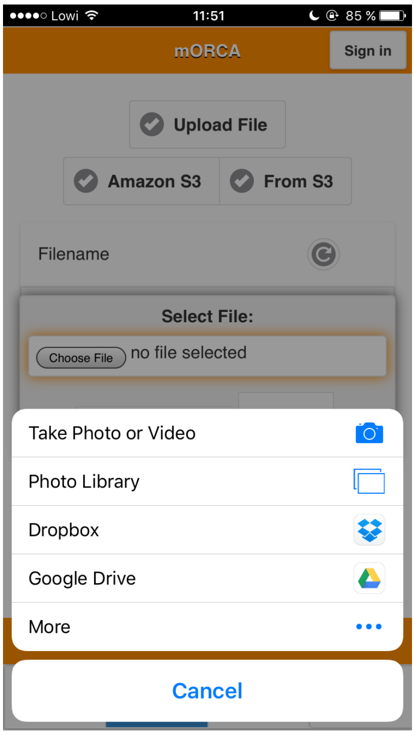

Also an option to import files from AmazonS3 is provided. At first, the credentials for the account should be setted in the popup menu. Then the files are listed in other popup and can be imported to our server-side file system.

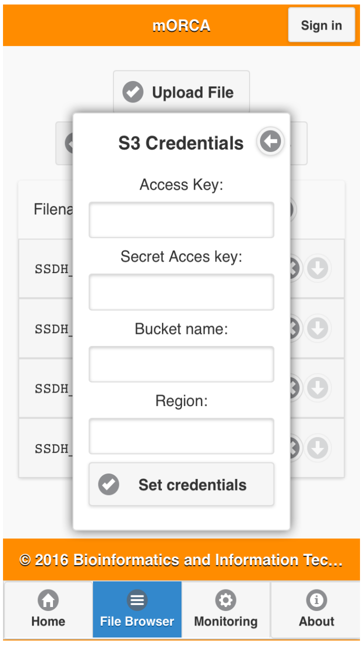

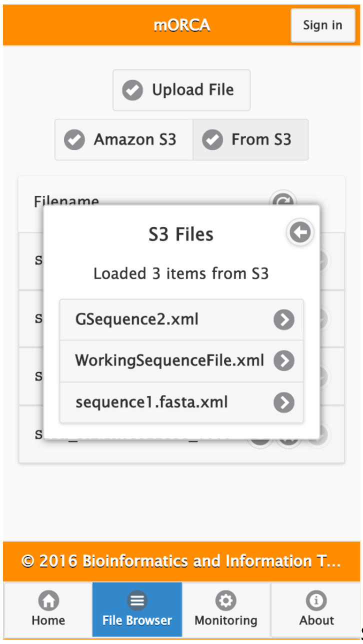

s

7. File Browsing

As your Cloud file storage gets cluttered with previously saved files, you can browse and delete the list of all of them.

Simply click the **File Browser** button at the bottom of the main screens and you will be listed all your files to deal with them.

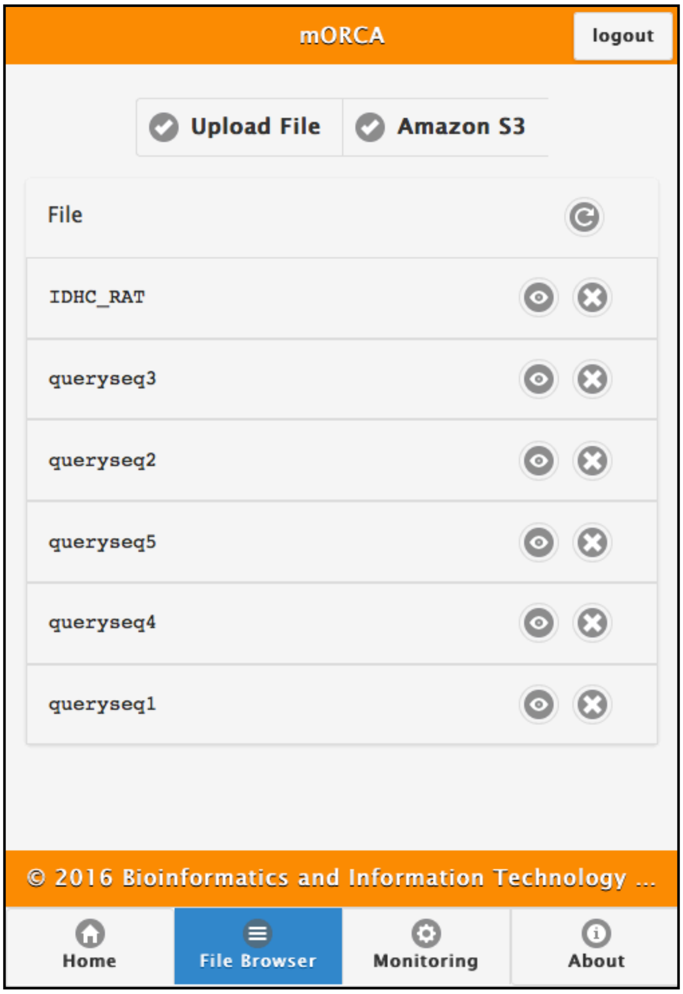

8. References

- S. Diaz-del-Pino, J.Karlsson, J. Falgueras, O.Trelles; Mobile Access to On-line Analytic Bioinformatics Tools, CL, Bioinformatics and Biomedical Engineering: Third International Conference, IWBBIO 2015, Granada, Spain, April 15-17, 2015. Proceedings, Part II, 555--565, Springer International Publishing
- Noura Chelbat, S. Diaz-del-Pino, Johan Karlsson, Oswaldo Trelles, Juan Falgueras; Usability tests on bioinformatics mobile applications, Jornadas de Bioinformatica, Sevilla, Septiembre 2014
- S. Diaz-del-Pino, O. Torreño, J. Karlsson, O.Trelles, J. Falgueras; Bioinformatics with mobile devices, Jornadas de Bioinformatica, Sevilla, Septiembre 2014
- S. Diaz, J. Karlsson, J. Falgueras, O. Trelles; Mobile access to on-line analytic bioinformatics tolos. http://chirimoyo.ac.uma.es/bitlab/compartir/morca/morcaIWWBIO.pdf
- Sergio Díaz, Johan Karlsson, Juan Falgueras, Oswaldo Trelles; Bioinformatic universal service access usable for all. http://chirimoyo.ac.uma.es/bitlab/compartir/morca/posterECCB.pdf
- A Sergio Díaz, Óscar Torreño, Johan Karlsson, Juan Falgueras, Oswaldo Trelles; Bioinformatics with mobile devices. http://chirimoyo.ac.uma.es/bitlab/compartir/morca/usabilidadJBI.pdf

mORCA: Annex 1

Listing new repositories in mORCA

mORCA manages each repository as a HTML file with an unordered list inside the ‘data’ folder.

BioTools is a special case, because at the moment its API only allows to list the services but not to invoke them. This mainly because it doesn’t use a common interface for them, making it more like a directory of services. Anyway, we are going to use it as example.

In the case of BioTools, the first step was writing an AJAX petition to its API to get the service list in JSON. In the callback of the petition, with the service list obtained, we make a function to generate the HTML unordered list.

For each service we filter those that are accessible using SOAP, then we create a ‘list item’ (li) object with the name and the description of the service.

```
function loadBioToolsServiceList(){

  function endsWith(str, suffix) {
    return str.indexOf(suffix, str.length - suffix.length) !== -1;
  }

  $.ajax({
    url: "https://bio.tools/api/tool?format=json"
  }).then(function(data) {
    var list = '<ul data-role="listview" id="tree" data-inset="true">';
    for (var i = 0; i < data.length; i++) {
      var service = data[i];
      var interface = service.interface;
      for (var j = 0; j < interface.length; j++){
        if(interface[j].interfaceType === 'SOAP WS' &&
            (typeof interface[j].interfaceSpecURL !== 'undefined' || endsWith(service.homepage,".wsdl"))) {
          list += '<li>' +
                  '<a href="#"><img src="img/serviceicon.png" alt="'+service.name+'">'+
                  '<h2>'+service.name+'</h2>'+
                  '<p>'+service.description+'</p></a></li>'
        }
      }
    }
  });
}
```

Figure 1. The AJAX petition and the callback to build the HTML list.

In the case of BioCatalogue, that doesn’t have an API available, they provide a JSON file that we must catch from an URL and then process it in the same way that before:

```
function listBiocatalogue() {

  $.ajax({
    url: 'getJSON.php',
    success: function(data) {
      data = jQuery.parseJSON(data);
      data = data.search.results;
      var list = '<ul data-role="listview" id="tree" data-inset="true">';
      for (var i = 0; i < data.length; i++) {
        var service = data[i];
        list += '<li>' +
            '<a href="#"><img src="img/serviceicon.png" alt="'+service.name+'">'+
            '<h2>'+service.name+'</h2>'+
            '<p>'+service.resource+'</p></a></li>'
        }
    }
  });
}
```

Figure 2. The same method applied to the Bio catalogue information.

After the lists are stored in the data folder, an option should be added to the select in the index page allowing the user to select them.

```
<!-- Repository Select -->
<div id="divSelectRepository" data-role="content" class="ui-field-contain" style="align-self: center">
    <label for="repository">Select repository: </label>
    <select id="selectRepository" name="repository" idname="select-choice-a" id="repository"
            data-native-menu="false" onchange="changeRepository();" data-mini="true">
        <option value="MrSymbiomath" selected="selected">Bitlab</option>
        <option value="INB">INB</option>
        <option value="Biotools" onclick='changeRepository("Biotools")'>Biotools</option>
        <option value="Biocatalogue" onclick='changeRepository("Biocatalogue")'>Biocatalogue</option>
    </select>
</div>

<div id="elixirlogodiv">
    <img src="img/logo.png" alt="MrSymbiomath" class="logo">
</div>

</div>
```

Figure 3. The select menu with the added options for the new repositories.
