# Supplementary material for: mORCA: ubiquitous access to life science web services
Source: BMC Genomics. 2018 Jan 16;19:56. doi: 10.1186/s12864-018-4439-x (PMC5771032; doi:10.1186/s12864-018-4439-x)
Supplement: Supplementary file 3 — Guided Exercise. The exercises described in the manuscript, step by step with screenshots and results. (PDF 2767 kb) [file 12864_2018_4439_MOESM3_ESM.pdf]

Mobiles: Ubiquitous access to life science Web Services

Sergio Diaz-del-Pino[1], Oswaldo Trelles[1] and Juan Falgueras[2,*]

{sergiodiazdp, ortrelles, juanfc}@uma.es

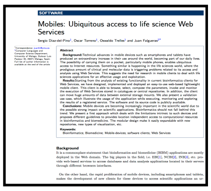

SOFTWARE

Mobiles: Ubiquitous access to life science Web Services

Sergio Diaz-del-Pino[1], Oscar Torreno[1], Oswaldo Trelles[1] and Juan Falgueras[2*]

*Correspondence: juanfc@uma.es
[2]Computer Languages and Computer Science Department, University of Malaga, Bulevar Luis Pasteur 35, 29071 Malaga, Spain
Full list of author information is available at the end of the article

Abstract

Background: Technical advances in mobile devices such as smartphones and tablets have produced an extraordinary increase in their use around the world, becoming part of our daily lives. The possibility of carrying them on a pocket, particularly mobile phones, enables ubiquitous access to Internet resources. Something similar is happening in the life sciences world, where the prodigious amount of clinical and molecular data is triggering problems related to its access and analysis using Web Services. This suggests the need for research in mobile clients to deal with life sciences applications for an effective usage and exploitation.

Results: Starting from the analysis of existing functionality in current bioinformatics clients for Web Services, we have designed, implemented and deployed an easy-to-use web-based lightweight mobile client. This client is able to browse, select, compose the parameters, invoke and monitor the execution of Web Services stored in catalogues or central repositories. In addition, the client can move huge amounts of data between external storage mounts. We also present a validation use case, which illustrate the usage of the application while executing, monitoring and exploring the results of a registered service. The software and its source code is publicly available.

Conclusions: Mobile devices are becoming increasingly important in the scientific world due to the possible strong impact on scientific applications. Bioinformatics should not fall behind this trend. We present a first approach which deals with the limitations intrinsic to such devices and proposes different guidelines to provides location independent access to computational resources in bioinformatics and biomedicine. The modular design make it easily expandable with new repositories, new types of visualization, etc.

Keywords: Bioinformatics; Biomedicine; Mobile-devices; software clients; Web Services

Background

It is a commonplace statement that bioinformatics and biomedicine (BIBM) applications are mostly deployed in the Web domain. The big players in the field; i.e. EBI[1], NCBI[2], INB[3], etc. provide web-based services to access databases and data analysis applications located in their servers through different browsers interfaces.

On the other hand, the rapid proliferation of mobile devices, including smartphones and tablets, makes the development of new clients for these devices to access scientific applications an ur-

(1) Computer Architecture Department
(2) Computer Languages and Computer Science Department
University of Malaga, Boulevard Luis Pasteur 35, 29071 Malaga, Spain
(*) Corresponding author

Document submitted to 'BMC Bioinformatics'; in review process.

---

mORCA

Guided Exercise

This document is the *Guided Exercise* presented in the section Results of the paper

http://chirimoyo.ac.uma.es/morca

Content

1. Getting started
2. First exercise: Retrieving a sequence (from login to service execution)
3. Second exercise: Running Blast
4. Third exercise: running a workflow
5. References

Contact: sergiodiazdp@uma.es (Sergio)

ortrelles@uma.es

November 2016

1. Getting started

Application access: mORCA is available in the App store by Apple and Play Store by Google. It is also available as web-app in the URL: http://chirimoyo.ac.uma.es/morca/app/

This document presents a quick survey to learn how to launch an application and do not describe the functionality available in each screen which is managed in the "mORCA user Guide". For this **additional information**, please refer to http://chirimoyo.ac.uma.es/morca

Main screen

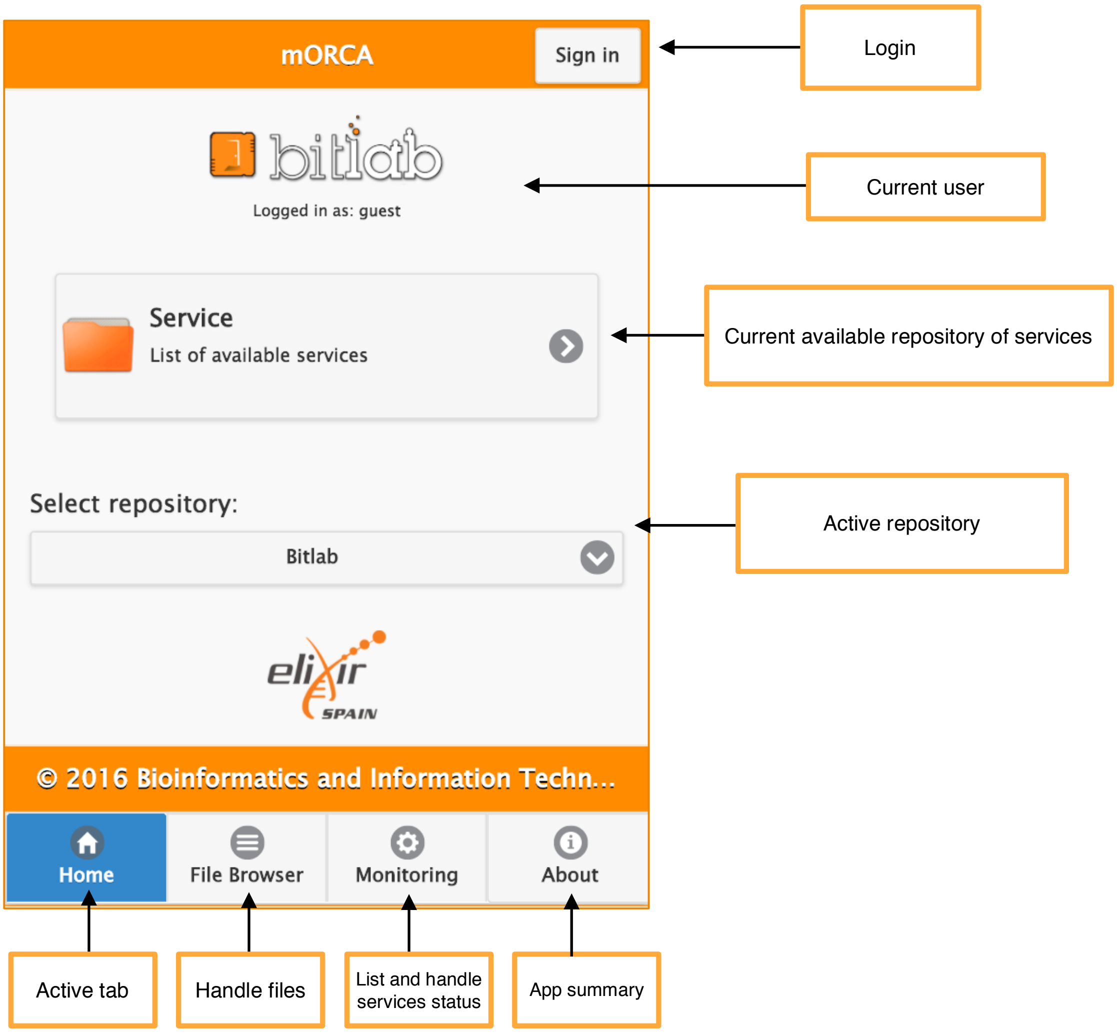

2. E1: First exercise: retrieving a sequence (from login to service execution)

This exercise is aimed to show the steps needed to complete the execution of a bioinformatics service: retrieving a biological sequence by its ID.

This exercise also includes the login, repository selection and browsing the catalogue actions.

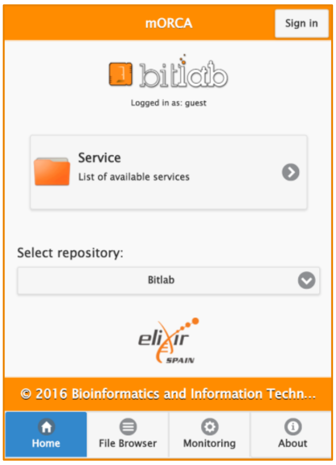

E1.a Launching the application. This is the first screen you will get when contact with mORCA

The application is available at http://chirimoyo.ac.uma.es/morca/app/

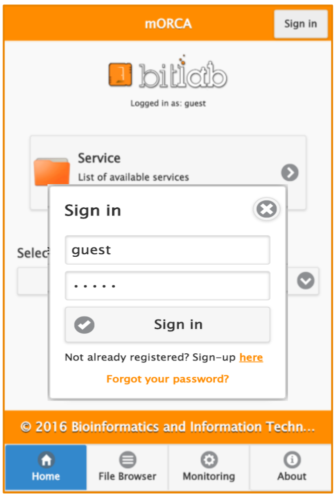

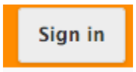

E1.b Login: Use your user / password credentials as the first step to get access to services.

A guest / guest generic set of credentials is available for testing purposes. //*** The system is prepared to request your password

Forgot your password?

Please send an email to the system administrator: sergiodiazdp@uma.es or to ortrelles@uma.es with your user name. A new password will be sent to your registered email

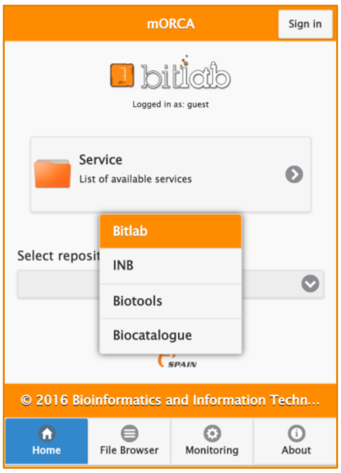

E1.c Choosing the repository of services.

This is only an informative step. There already are several available repositories, but the exercise will be conducted over the “by default” repository –BITLAB.

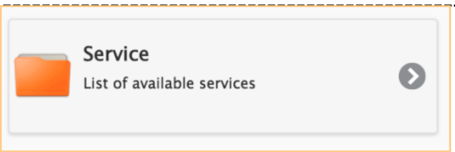

E1.d In the main screen the first level of the service catalogue is displayed. This button gives access to the full list of services

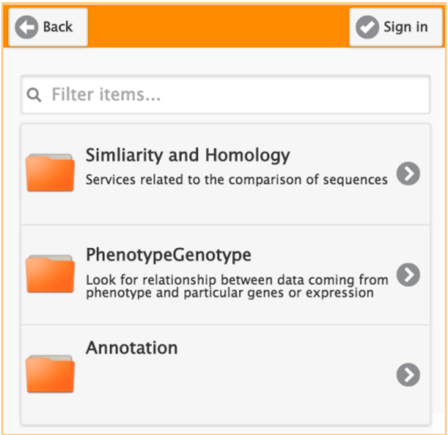

E1.e The second level of the services list. Choose “Similarity and Homology” category.

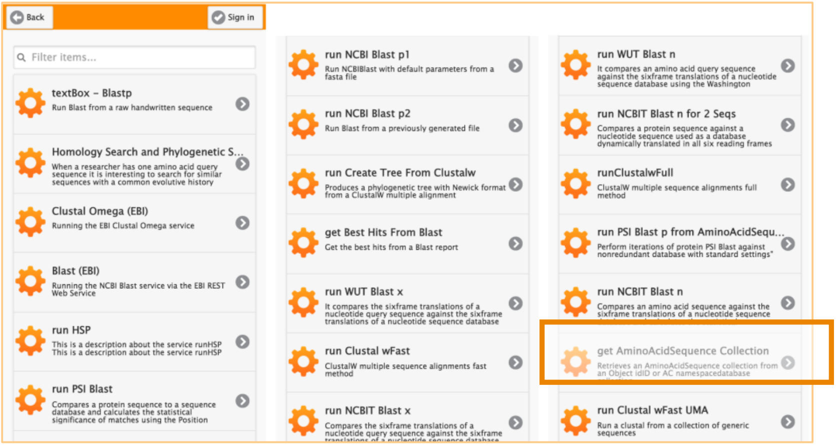

E1.f Currently this category is populated with a large list of services. Here a sketch composition. Choose the “get Amino-acid Sequence” service.

(See below for a searching procedure)

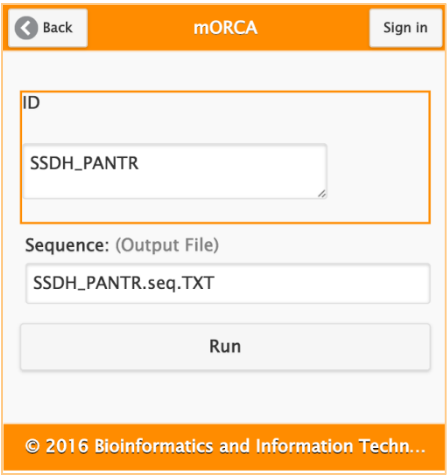

E1.g Once the service is invoked a new screen request the service parameters. In this case, a protein ID and an output filename is needed. We use `SSDH_PANTR` as sequence ID in this exercise, and will name `SSDH_PANTR.seq.TXT` the output file.

Use the “Run” button to launch the service.

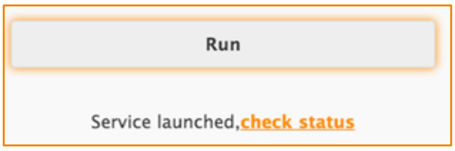

E1.g.1 Just after launching the service a message to follow-up the execution is displayed. The user will be conducted to the “monitoring” window also automatically.

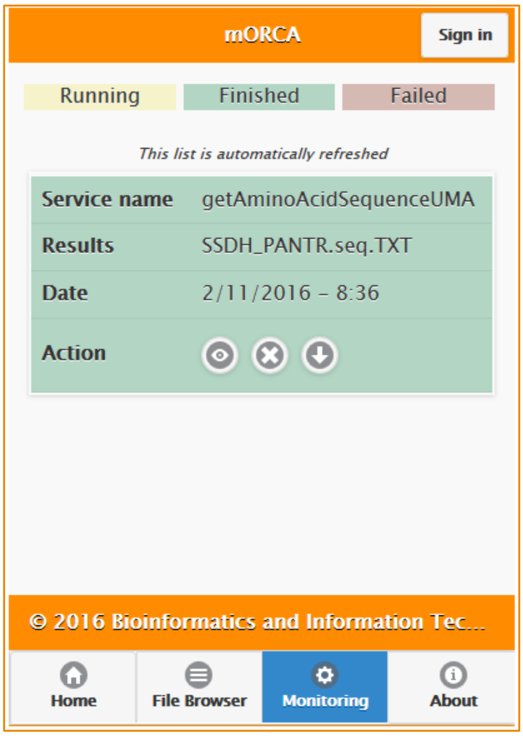

E1.h Service monitoring. Colors represent the status of the service, Running, Finished or Failed. In the event of success the info of the service is displayed. At this point, the user can

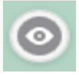

Visualize the result file content

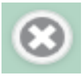

Delete the service status from the monitoring list.

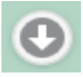

Download the result file.

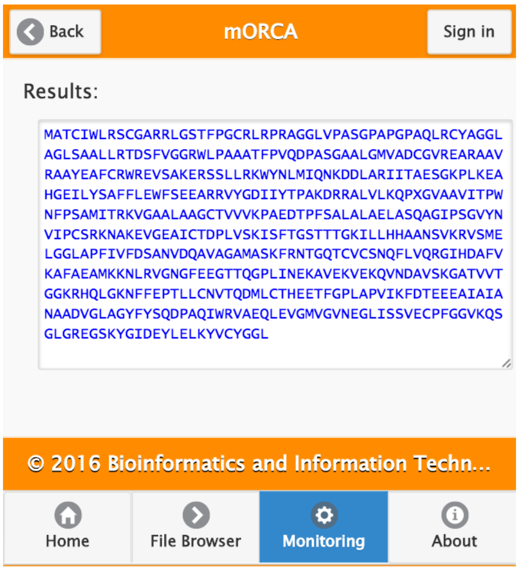

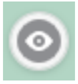

E1.i Visualization (text files)

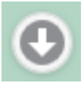

E1.j File download

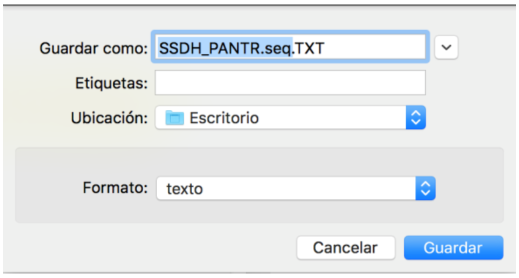

3. E2: Second exercise: Running Blast

Press the Back button at the top left corner: 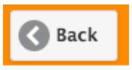

Filter the long list with the word “Blast” and pick “**run NCBI Blast p1**”

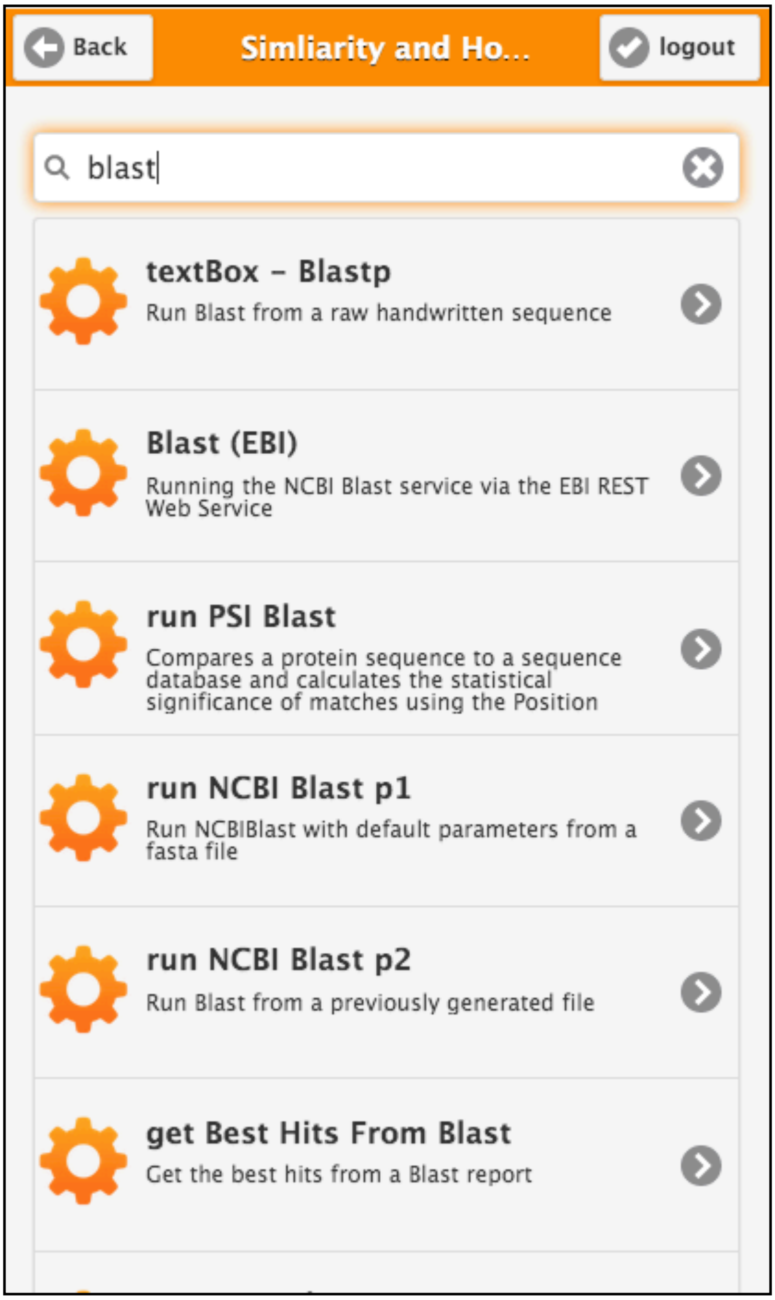

In the Blast Dialog

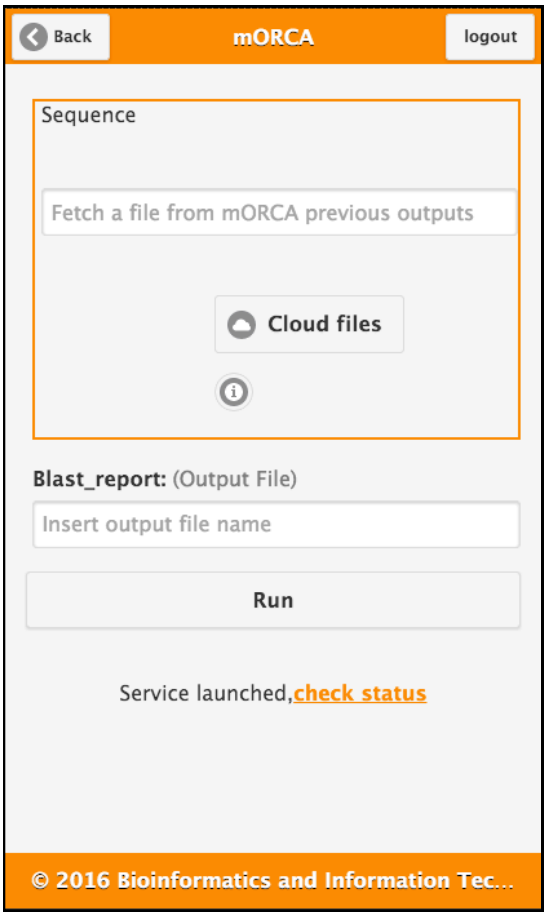

Press the “**Cloud files**” button to pick our previously downloaded **SSDH_PANTR** sequence

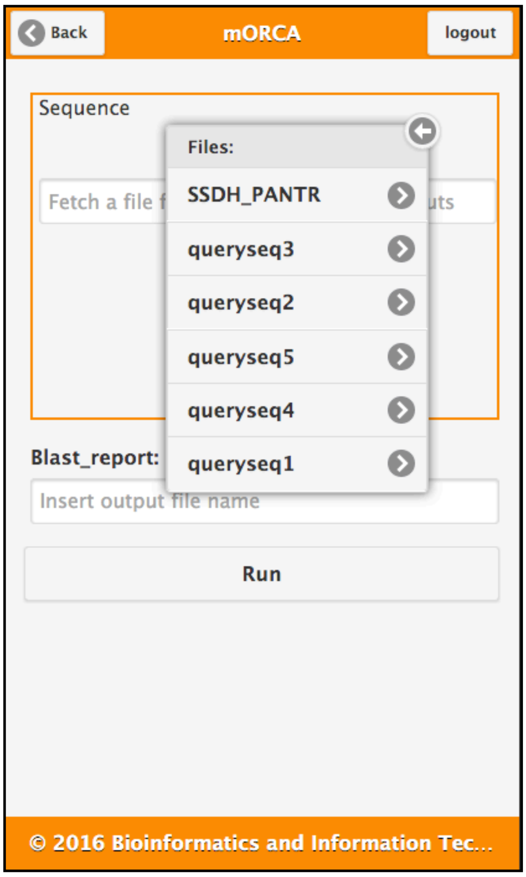

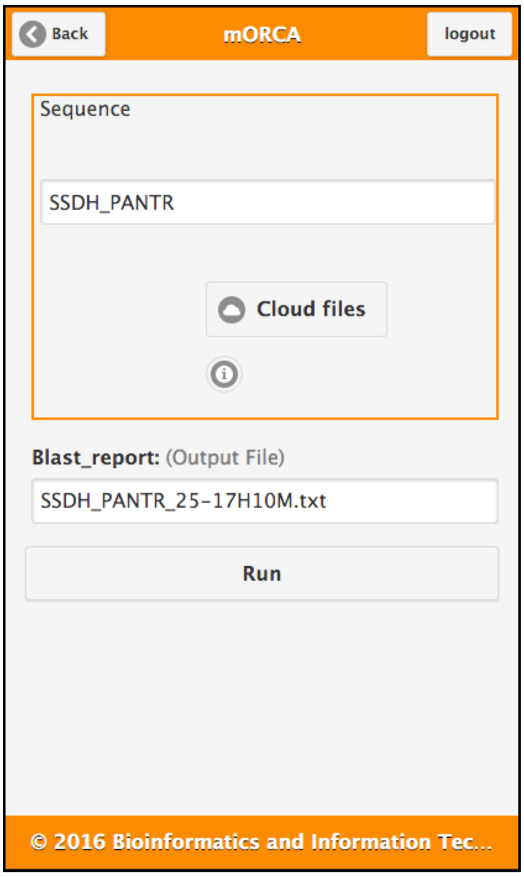

You can leave the default output file name, as it safely does not coincide with any other in you

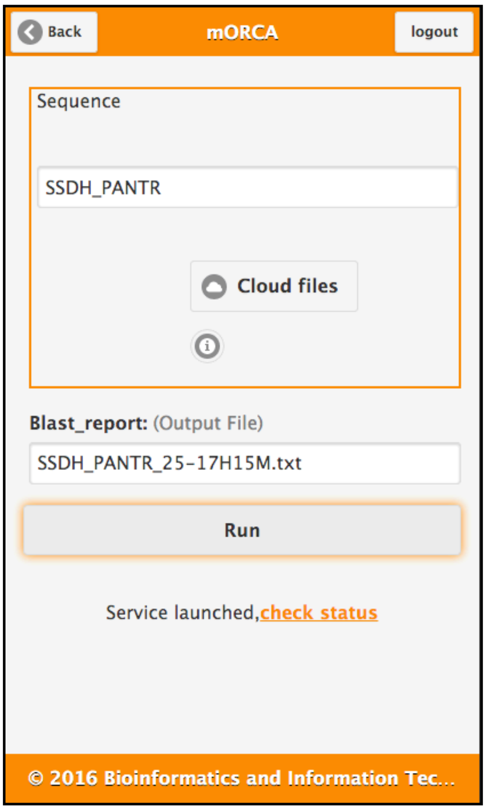

repository. **Run** the service:

You can see a text with a link to the **check status** dialog has appeared. Touch it right here and you will see your process at the top, and its status in green color (or wait until it changes to green):

Click on the **Action** button with an eye of the top and in the resulting text you have the answer: *DAVD_PSEAE* as its most important alignment.

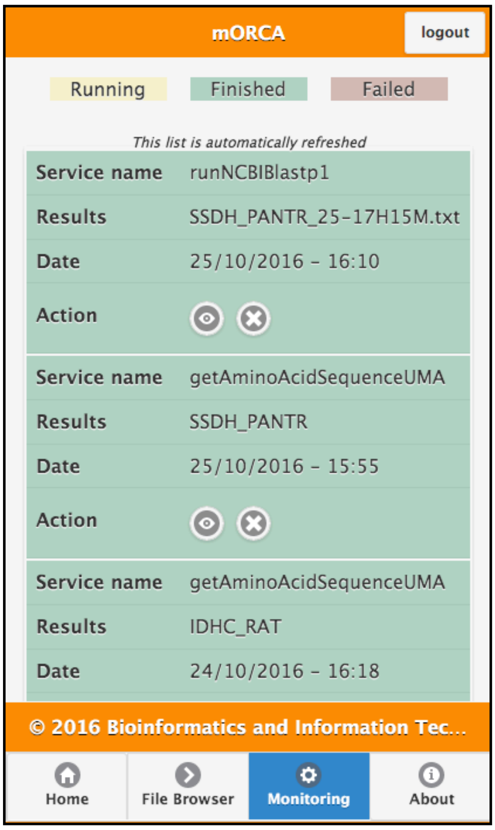
mORCA
logout
Running
Finished
Failed
This list is automatically refreshed
Service name runNCBIBlastp1
Results SSDH_PANTR_25-17H15M.txt
Date 25/10/2016 - 16:10
Action
Service name getAminoAcidSequenceUMA
Results SSDH_PANTR
Date 25/10/2016 - 15:55
Action
Service name getAminoAcidSequenceUMA
Results IDHC_RAT
Date 24/10/2016 - 16:18
© 2016 Bioinformatics and Information Tec...
Home
File Browser
Monitoring
About

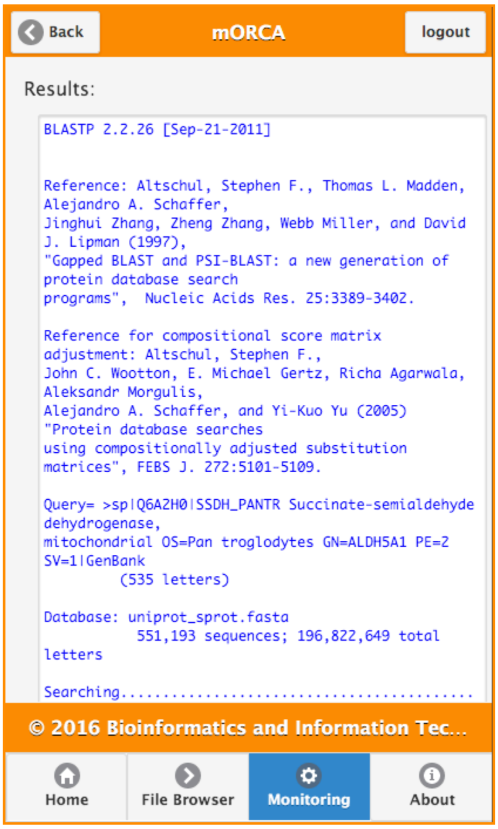
Back
mORCA
logout
Results:
BLASTP 2.2.26 [Sep-21-2011]
Reference: Altschul, Stephen F., Thomas L. Madden, Alejandro A. Schaffer, Jinghui Zhang, Zheng Zhang, Webb Miller, and David J. Lipman (1997), "Gapped BLAST and PSI-BLAST: a new generation of protein database search programs", Nucleic Acids Res. 25:3389-3402.
Reference for compositional score matrix adjustment: Altschul, Stephen F., John C. Wootton, E. Michael Gertz, Richa Agarwala, Aleksandr Morgulis, Alejandro A. Schaffer, and Yi-Kuo Yu (2005) "Protein database searches using compositionally adjusted substitution matrices", FEBS J. 272:5101-5109.
Query= >sp|Q6A2H0|SSDH_PANTR Succinate-semialdehyde dehydrogenase, mitochondrial OS=Pan troglodytes GN=ALDH5A1 PE=2 SV=1|GenBank
(535 letters)
Database: uniprot_sprot.fasta
551,193 sequences; 196,822,649 total letters
Searching.......................................
© 2016 Bioinformatics and Information Tec...
Home
File Browser
Monitoring
About

4. E3: Third exercise: running a workflow (Homology search and Phylogenetic Study)

This exercise is aimed to show the steps needed to complete the execution of a bioinformatics workflow. This workflow is used to ilustrate the execution of the main document. This workflow is composed of the following services:

A. **Get amino acid sequence**: Retrieves an amino acid sequence from the Uniprot database using the sequence ID.

B. **Run EBI Blast**: It runs a BlastP (homology search using UnitprotKb –a protein database) in the EBI server with the retrieved sequence. This is an external EBI service.

C. **Get best hits from Blast**: It extracts the most related sequences to the Blast report query using an E-value threshold. This value is set by default in this workflow to 0.02 (the service by itself has the possibility to change it). The output is a collection of sequences each with an ID/AC and namespace.

D. **Get Amino Acid Sequence collection**: This service returns a set of amino acid sequences corresponding to the given ID/Namespace pairs. The output is a list with the retrieved sequences

E. **Run ClustalW**: It performs a multiple sequences alignment using the ClustalW algorithm with the set of the retrieved sequences. The output of this service is the sequence alignment from the sequence similarity among the analysed sequences.

F. **Run Create Tree from ClustalW**: Finally, the relations obtained with the multiple alignment are used to build a dendrogram tree with Newick format.

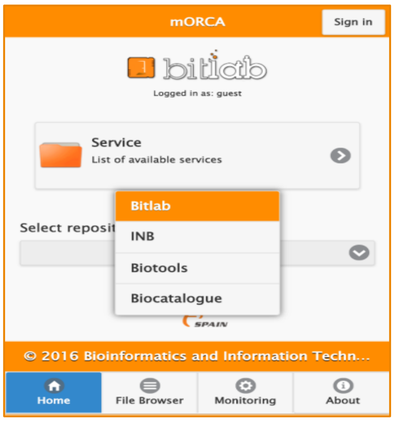

E3A.a Launching the application: This is the first screen you will get when contact with mORCA

The application is available at http://chirimoyo.ac.uma.es/morca/app/

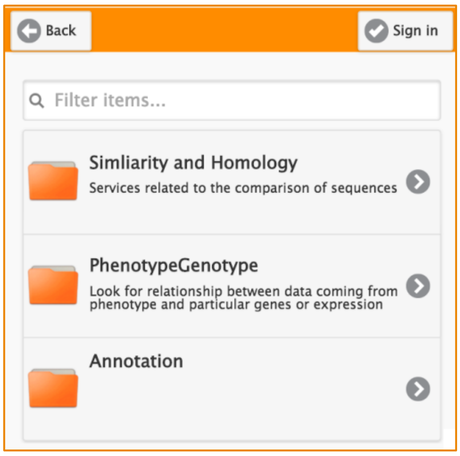

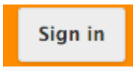

E3A.b Login: Use your user / password credentials as the first step to get access to services.

A guest / guest generic set of credentials is available for testing purposes.

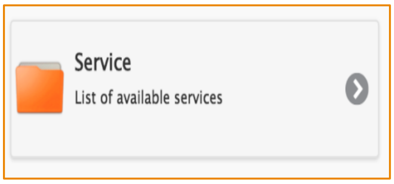

E3A.c Choosing the repository of services.

This is only an informative step. There already are several available repositories, but the exercise will be conducted over the “by default” repository –BITLAB.

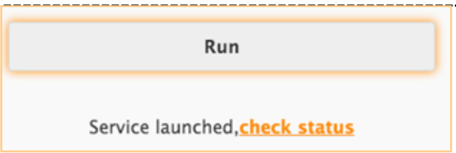

E3A.d In the main screen the first level of the service catalogue is displayed. This button gives access to the full list of services.

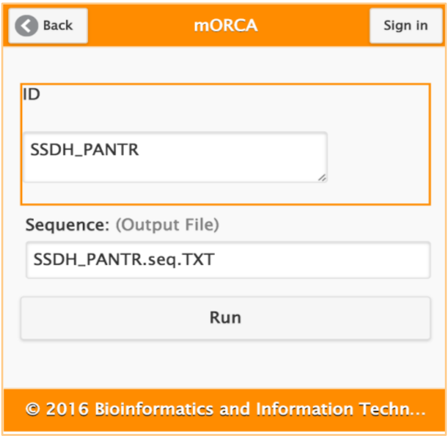

E3A.e The second level of the services list. Choose "Similarity and Homology" category.

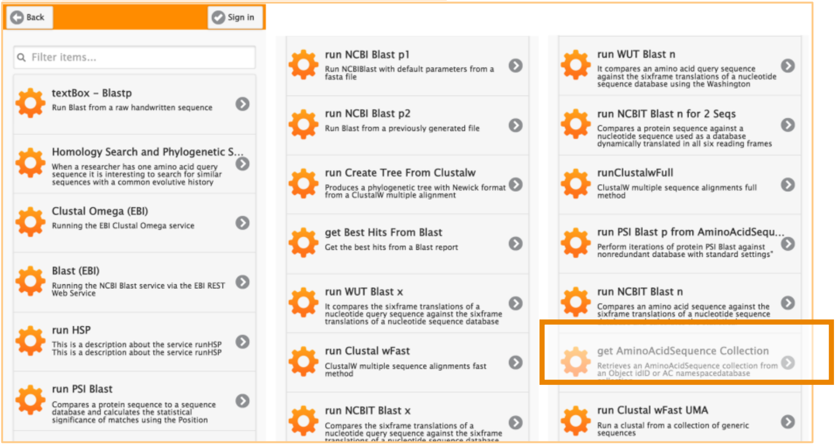

E3A.f Currently this category is populated with a large list of services. Here a sketch composition. Choose the "get Amino-acid Sequence" service

(see below for a searching procedure)

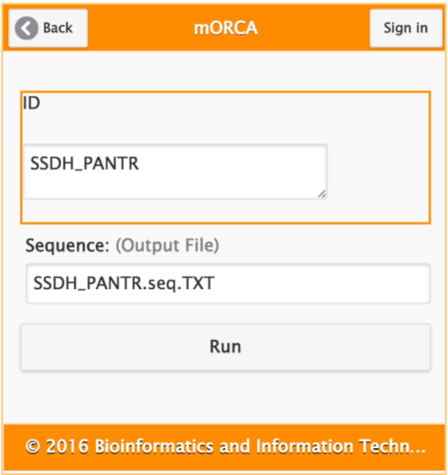

E3A.g Once the service is invoked a new screen request the service parameters. In this case, a protein ID and a output filename is needed. We use **`SSDH_PANTR`** as sequence ID in this exercise, and will name **`SSDH_PANTR.seq.TXT`** the output file.

Use the "Run" button to launch the service

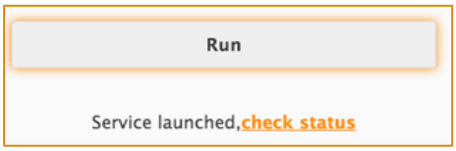

E3A.g.1 Just after launching the service a message to follow-up the execution is displayed. The user will be conducted to the "monitoring" window also automatically

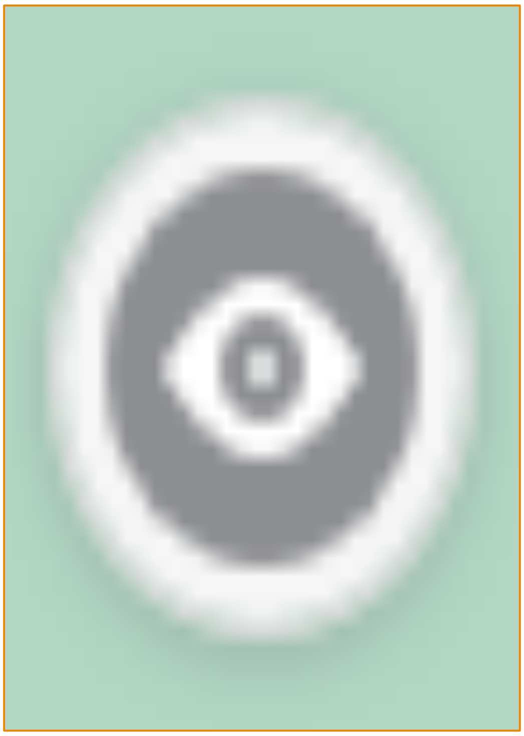

E3A.h Service monitoring. The colours represent the status of the service, Running, Finished or Failed. In the event of success the info of the service is displayed. At this point, the user can

E3B. Run EBI Blast

Press the Back button at the top left corner: 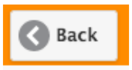

filter the long list with the word “blast” and pick “**run NCBI Blast p1**”

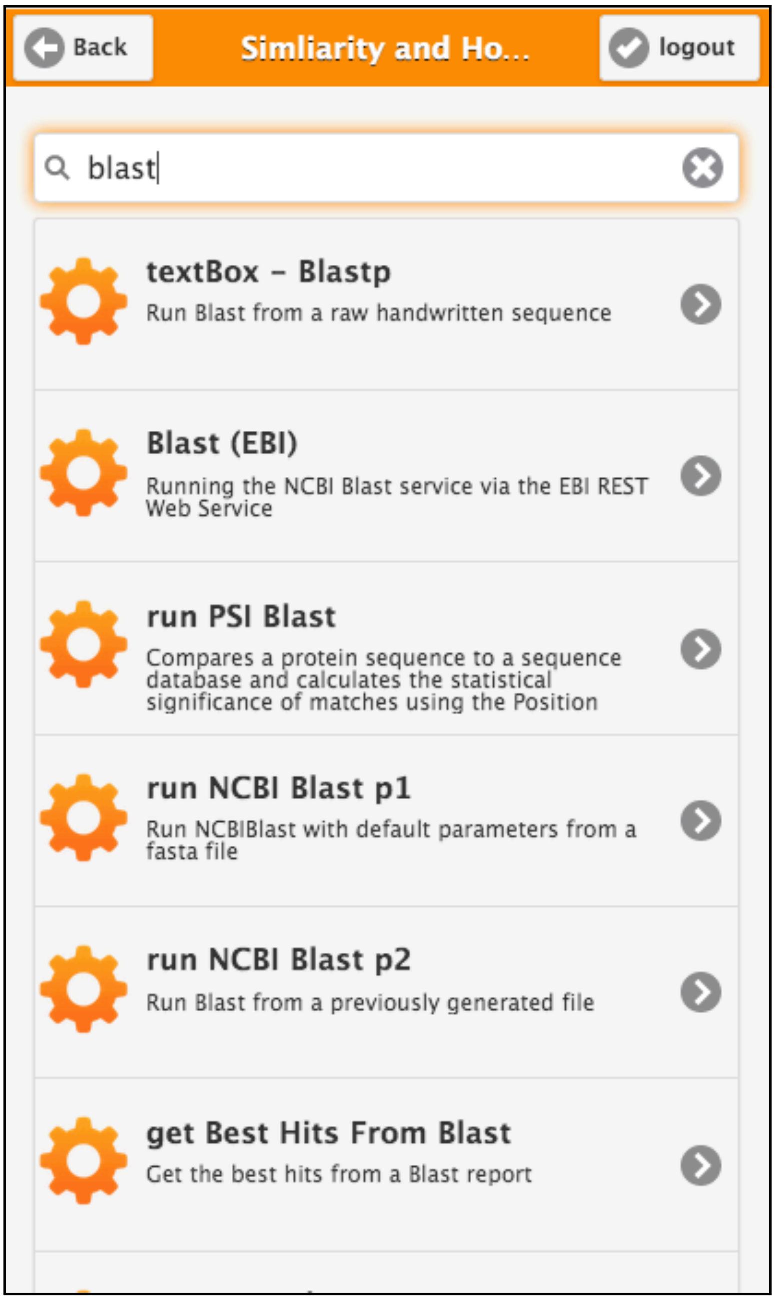

In the Blast Dialog

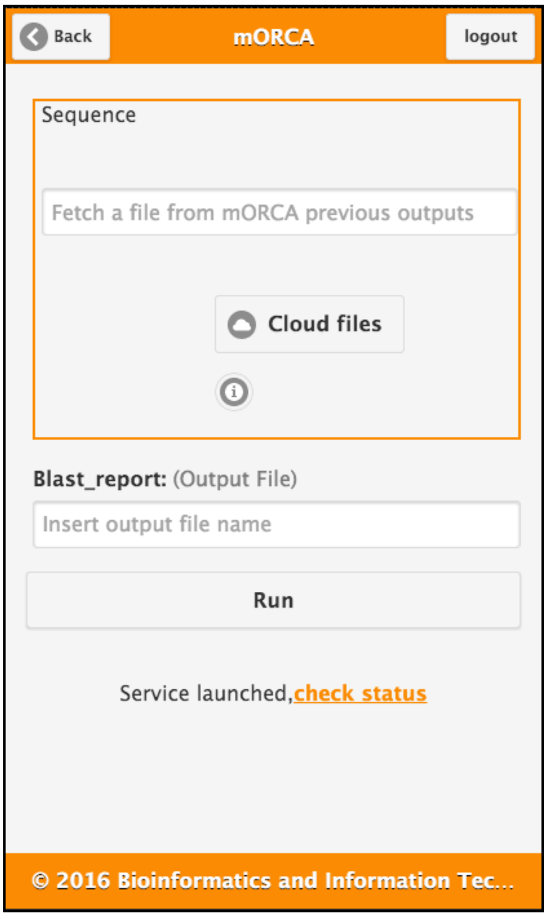

Press the "**Cloud files**" button to pick our previously downloaded **SSDH_PANTR** sequence

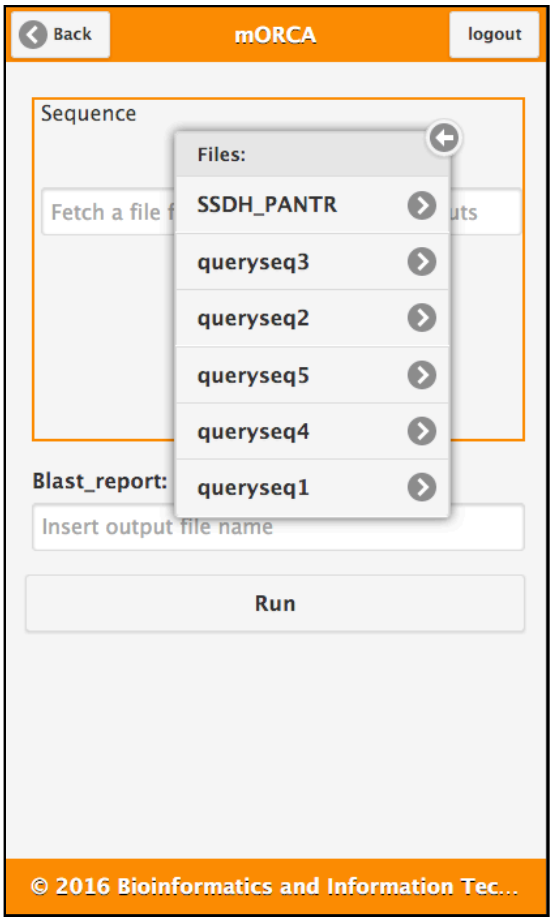

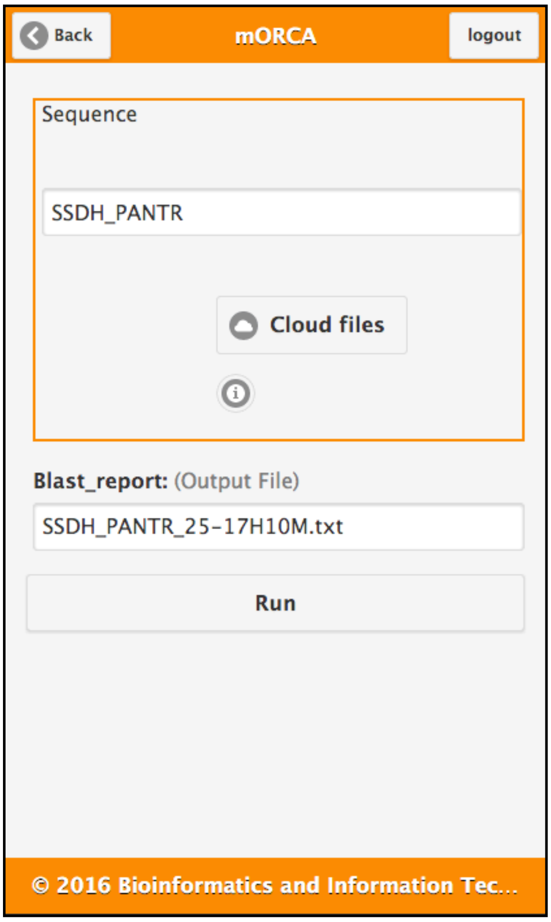

You can leave the default output file name, as it safely does not coincide with any other in you repository. So **Run** the service:

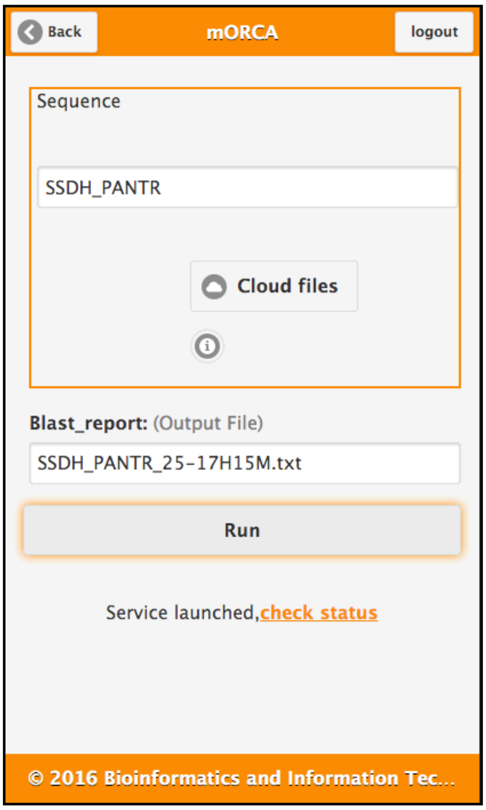

you can see a text with a link to the **check status** dialog has appeared. Touch it right here and you will see your process at the top, and its status in green color (or wait until it changes to green):

Click on the **Action** button with an eye of the top and in the resulting text you have the answer: DAVD_PSEAE as its most important alignment.

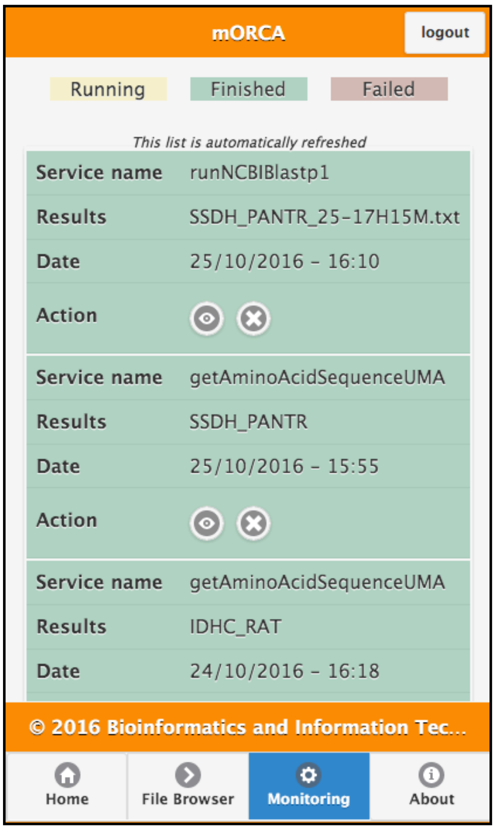

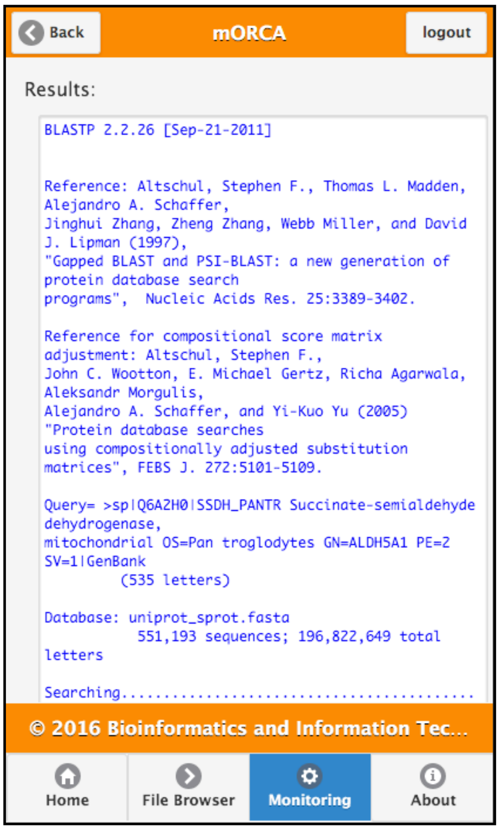

E3C. Get best hits from Blast

Press the Back button at the top left corner:

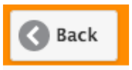

filter the long list with the word “hit” and pick “**get Best Hits From Blast**” and choose the appropriate file from the Cloud

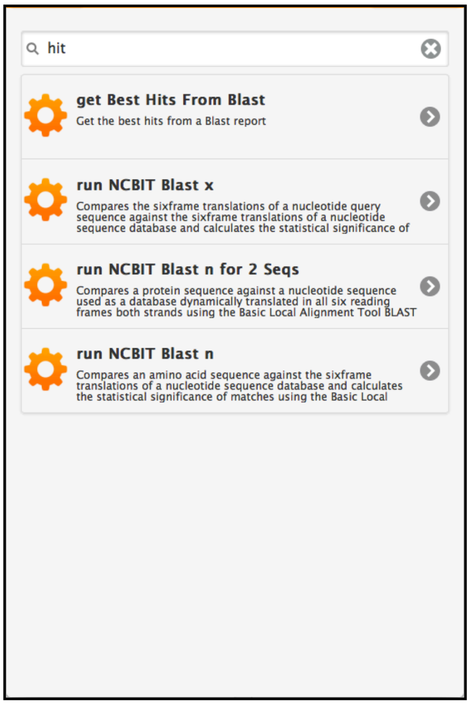

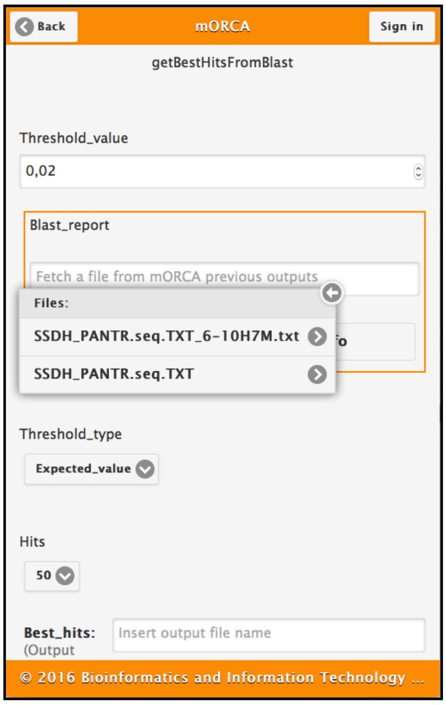

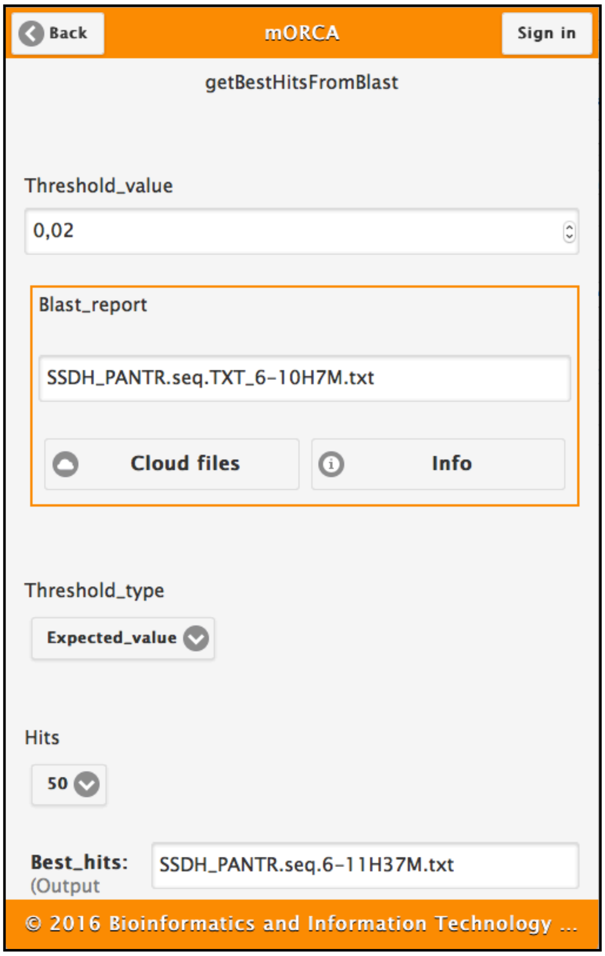

Once chosen, for this exercise, leave the default Output name as it is.

Scroll down to the Run button, and check the output.

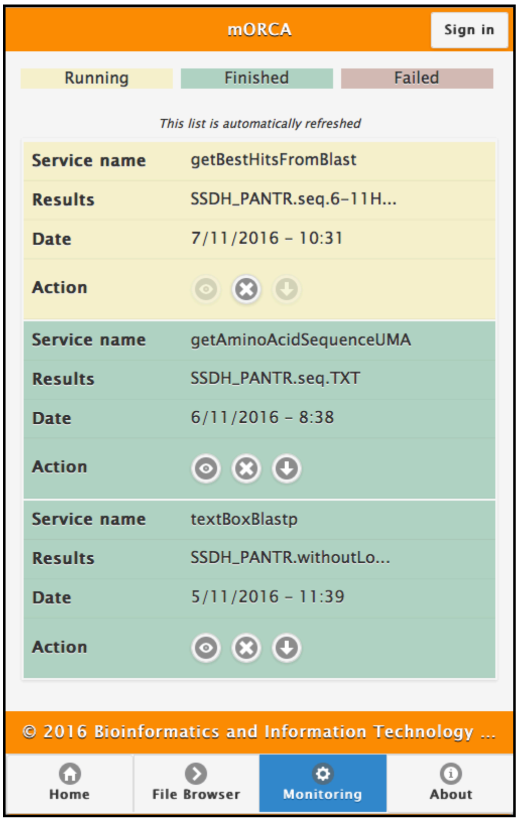

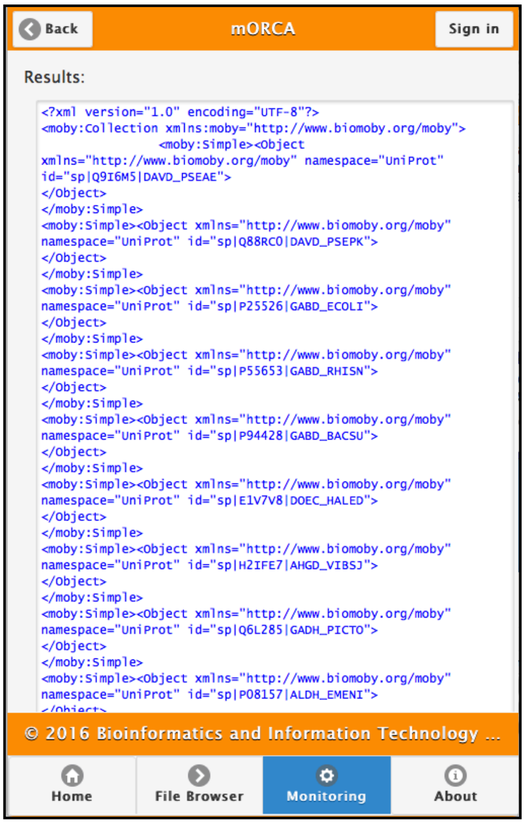

E3D. Get AminoAcidSequence Collection

Go back as usual, and filter “coll” to pick the “**get AminoAcidSequence Collection**”. Chose the previous output file and run it.

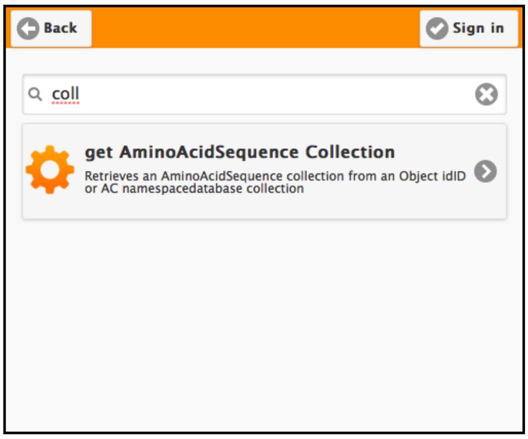

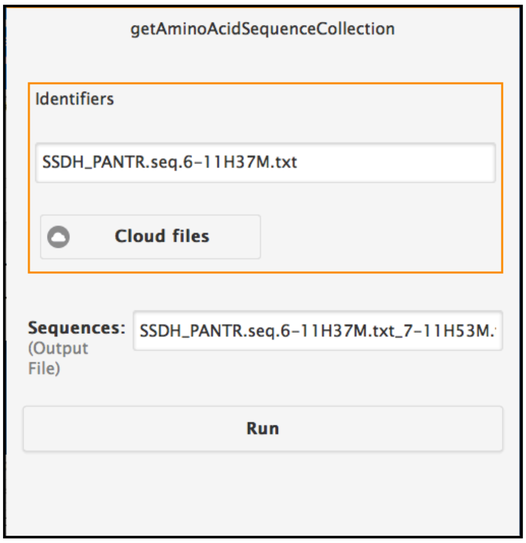

After running it and checkout you can see the list of relate retrieved sequences:

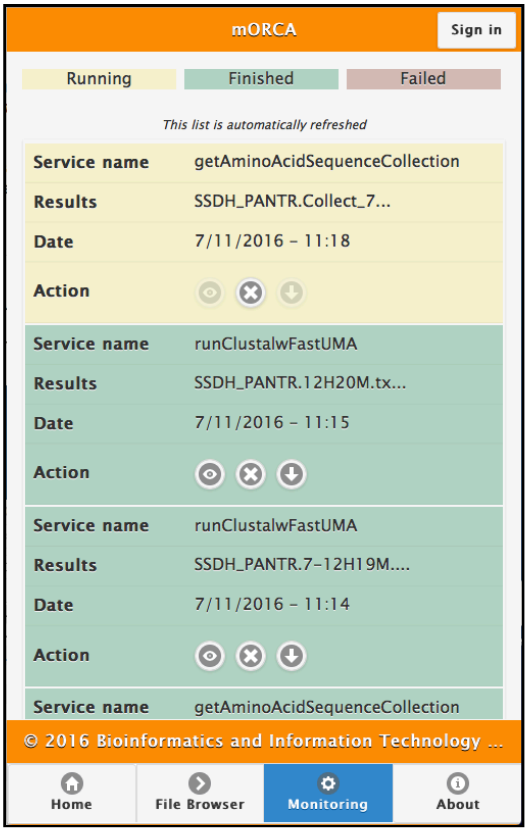

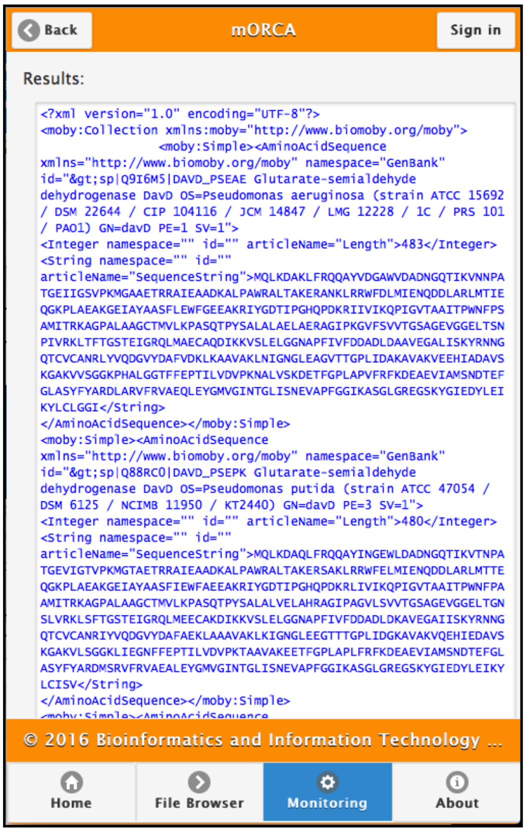

E3E. Run ClustalW

Go back as usual, and filter "cl" to pick the "**run Clustal wFast UMA**". Scroll down to choose the previous file from **Cloud files**.

After running it and checkout you can see the output of Clustal:

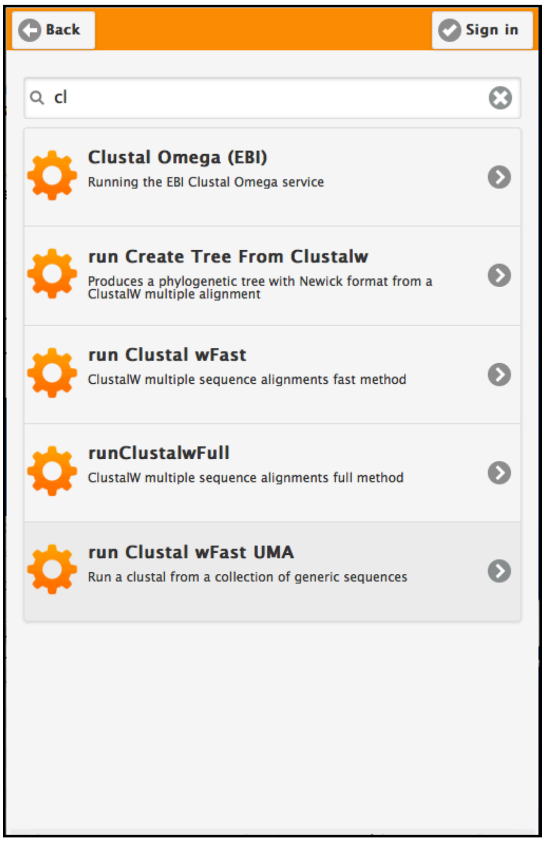

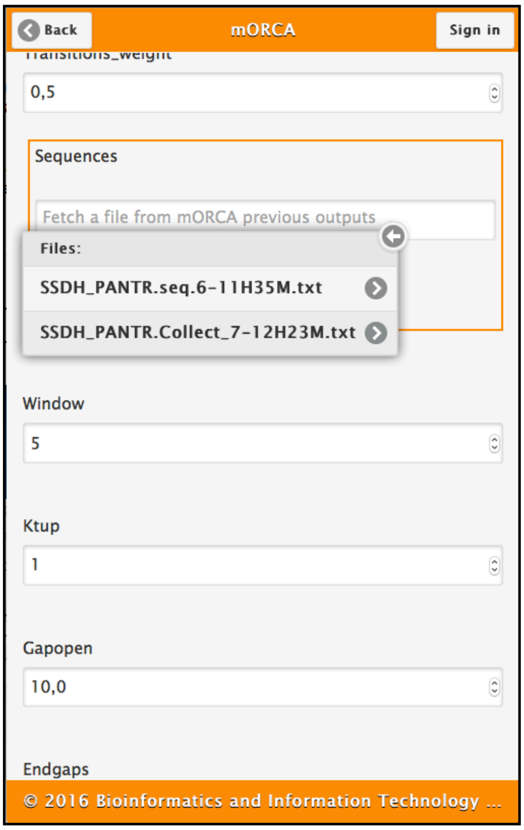

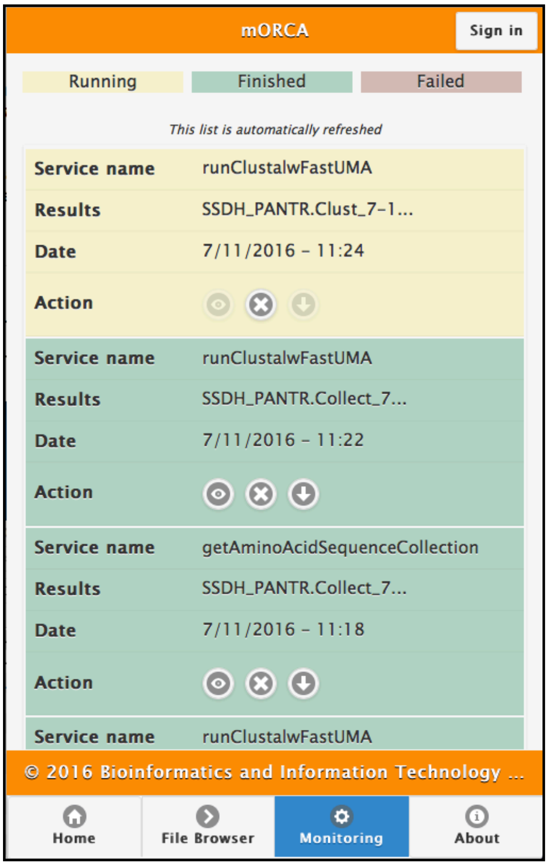

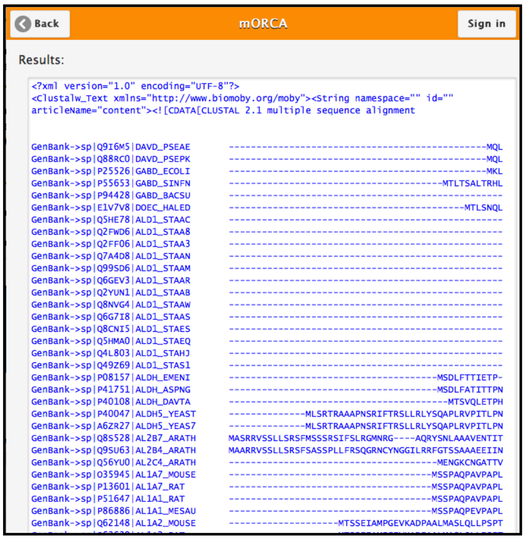

E3F. Run ClustalW

Go back as usual, and filter "tree" to pick the "**run Create Tree From Clustalw**". Scroll down to choose the previous file from **Cloud files**.

After running it and checkout you can see the Newick dendrogram

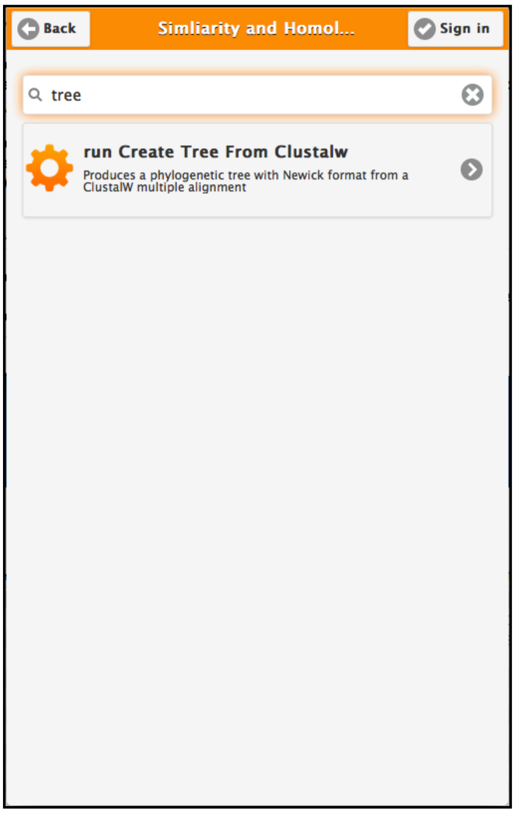

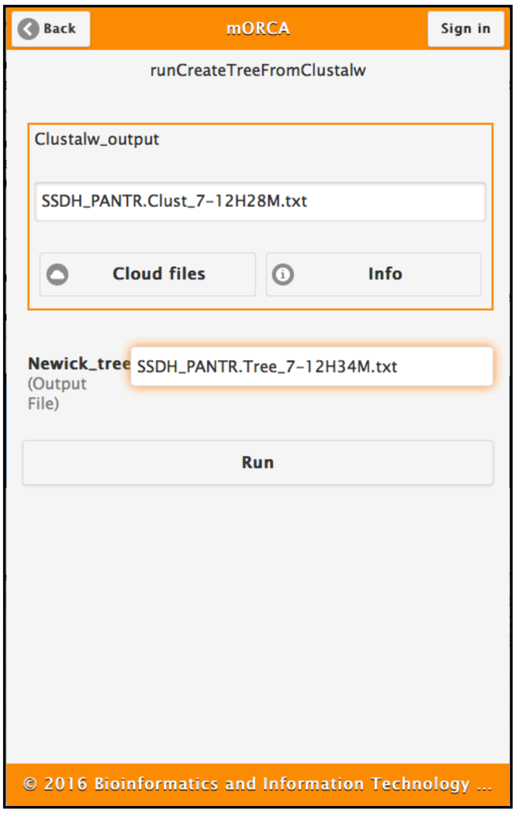

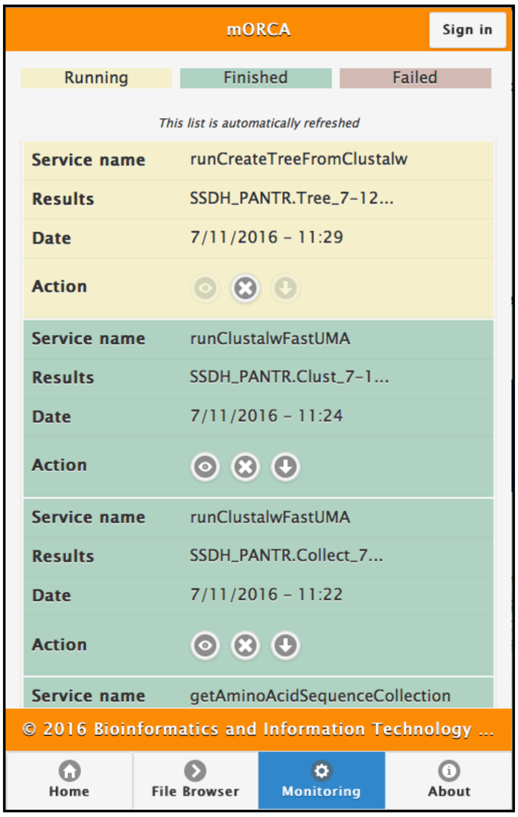

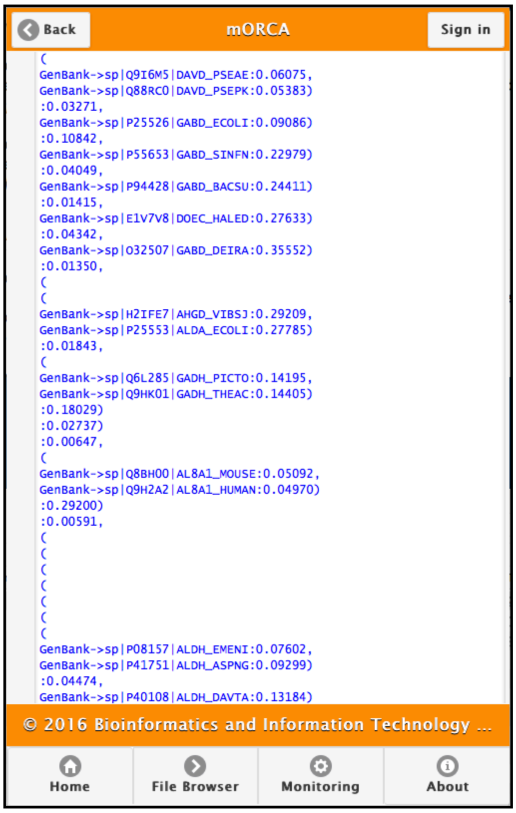

5. References:

- S. Diaz-del-Pino, J.Karlsson, J. Falgueras, O.Trelles; Mobile Access to On-line Analytic Bioinformatics Tools, CL, Bioinformatics and Biomedical Engineering: Third International Conference, IWBBIO 2015, Granada, Spain, April 15-17, 2015. Proceedings, Part II, 555--565, Springer International Publishing
- Noura Chelbat, S. Diaz-del-Pino, Johan Karlsson, Oswaldo Trelles, Juan Falgueras; Usability tests on bioinformatics mobile applications, Jornadas de Bioinformatica, Sevilla, Septiembre 2014
- S. Diaz-del-Pino, O. Torreño, J. Karlsson, O.Trelles, J. Falgueras; Bioinformatics with mobile devices, Jornadas de Bioinformatica, Sevilla, Septiembre 2014
- S. Diaz, J. Karlsson, J. Falgueras, O. Trelles; Mobile access to on-line analytic bioinformatics tolos. http://chirimoyo.ac.uma.es/bitlab/compartir/morca/morcaIWWBIO.pdf
- Sergio Díaz, Johan Karlsson, Juan Falgueras, Oswaldo Trelles; Bioinformatic universal service access usable for all. http://chirimoyo.ac.uma.es/bitlab/compartir/morca/posterECCB.pdf
- A Sergio Díaz, Óscar Torreño, Johan Karlsson, Juan Falgueras, Oswaldo Trelles; Bioinformatics with mobile devices. http://chirimoyo.ac.uma.es/bitlab/compartir/morca/usabilidadJBI.pdf
